# A SWATH-MS analysis of Myalgic Encephalomyelitis/Chronic Fatigue Syndrome peripheral blood mononuclear cell proteomes reveals mitochondrial dysfunction

**DOI:** 10.1186/s12967-020-02533-3

**Published:** 2020-09-24

**Authors:** Eiren Sweetman, Torsten Kleffmann, Christina Edgar, Michel de Lange, Rosamund Vallings, Warren Tate

**Affiliations:** 1 Department of Biochemistry, University of Otago, Dunedin, 9016 New Zealand; 2 Centre for Biostatistics, University of Otago, Dunedin, 9016 New Zealand; 3 Howick Health and Medical Centre, Auckland, 2014 New Zealand

**Keywords:** Myalgic encephalomyelitis, Chronic fatigue syndrome, Diagnostic biomarker, Mitochondria, Oxidative phosphorylation, Reactive oxygen species, Oxidative stress, Metabolism, Inflammation and immunity

## Abstract

**Background:**

Myalgic Encephalomyelitis/Chronic Fatigue Syndrome (ME/CFS) is a serious and complex physical illness that affects all body systems with a multiplicity of symptoms, but key hallmarks of the disease are pervasive fatigue and ‘post-exertional malaise’, exacerbation after physical and/or mental activity of the intrinsic fatigue and other symptoms that can be highly debilitating and last from days to months. Although the disease can vary widely between individuals, common symptoms also include pain, cognitive deficits, sleep dysfunction, as well as immune, neurological and autonomic symptoms. Typically, it is a very isolating illness socially, carrying a stigma because of the lack of understanding of the cause and pathophysiology.

**Methods:**

To gain insight into the pathophysiology of ME/CFS, we examined the proteomes of peripheral blood mononuclear cells (PBMCs) by SWATH-MS analysis in a small well-characterised group of patients and matched controls. A principal component analysis (PCA) was used to stratify groups based on protein abundance patterns, which clearly segregated the majority of the ME/CFS patients (9/11) from the controls. This majority subgroup of ME/CFS patients was then further compared to the control group.

**Results:**

A total of 60 proteins in the ME/CFS patients were differentially expressed (*P* < 0.01, Log_10_ (Fold Change) > 0.2 and < −0.2). Comparison of the PCA selected subgroup of ME/CFS patients (9/11) with controls increased the number of proteins differentially expressed to 99. Of particular relevance to the core symptoms of fatigue and post-exertional malaise experienced in ME/CFS, a proportion of the identified proteins in the ME/CFS groups were involved in mitochondrial function, oxidative phosphorylation, electron transport chain complexes, and redox regulation. A significant number were also involved in previously implicated disturbances in ME/CFS, such as the immune inflammatory response, DNA methylation, apoptosis and proteasome activation.

**Conclusions:**

The results from this study support a model of deficient ATP production in ME/CFS, compensated for by upregulation of immediate pathways upstream of Complex V that would suggest an elevation of oxidative stress. This study and others have found evidence of a distinct pathology in ME/CFS that holds promise for developing diagnostic biomarkers.

## Background

Myalgic Encephalomyelitis/Chronic Fatigue Syndrome (ME/CFS) is an illness characterized by debilitating fatigue lasting more than 6 months that is not alleviated by rest. The myriad of symptoms is exacerbated by physical or mental exertion. ME/CFS affects approximately 0.5−1% of the global population, all age groups and socioeconomic strata, but is more common in women (reports of this female bias vary, and range between a 2:1 to 6:1 female to male ratio) [1–3]. ME/CFS is a complex disease involving profound dysregulation of the central nervous system (CNS) and immune system, dysfunction of cellular energy metabolism and ion transport, and as well cardiovascular abnormalities [4]. The likelihood of a full recovery is poor; with as few as 6% of sufferers reported to return to their previous state of wellbeing [5, 6]. Flu-like and respiratory symptoms are common, and for a large subset of sufferers, the onset of the illness is sudden and is preceded by an acute viral-like infectious period. In many patients this is linked to an Epstein Barr infection and glandular fever [4]. There is speculation the current Covid-19 pandemic might see a significance increase in the incidence of ME/CFS, as reported after the original SARS epidemic [7]. Already, a high rate of post viral fatigue has been reported, and in some patients, this is likely to develop into long lasting ME/CFS.

Gradual onset of ME/CFS is also reported, with the ‘symptom complex’ developing over a period of several weeks or months [3]. Nevertheless, current diagnostic criteria describe ME/CFS as disabling physical and mental fatigue, usually of *acute* onset, which is significantly exacerbated by exercise and activity, and by mental or emotional exertion [3, 6]. A formal medical diagnosis can only be given after a process of exclusion of other well-established fatigue illnesses, and there is a range of self-reported symptoms that fit within one of several available widely defined sets of clinical criteria for ME/CFS [2, 8, 9]. There are as yet no agreed diagnostic markers or definitive clinical tests, the causative agent is not well understood, and the resulting disease pathophysiology is still ill-defined [10]. Immune dysfunction, chronic viral infection and, recently, metabolic and mitochondrial dysregulation have all been implicated as possible underlying consequences of ME/CFS that sustain the illness. We have also recently proposed that chronic neuro-inflammation involving the paraventricular nucleus of the hypothalamus could be a critical factor in sustaining ME/CFS [11]. In recent years ME/CFS research has shifted its focus from attempting to identify a universal ‘causative agent’ for the illness, to instead ascertaining the key affected physiology and biological pathways behind the ME/CFS symptom complex. High-throughput molecular analyses including cytokine production [12], metabolomics [13–16], the microbiome [17–19], epigenetics [20–23], transcriptome [24–26] and proteome investigations [27–32] have provided substantial evidence of immune/inflammation involvement, and, significantly, suggest there are deficits in energy metabolism and mitochondrial function in ME/CFS. While there have been inconsistencies in the evidence for mitochondrial dysfunction, a growing number of research papers are supportive of this hypothesis. Previous studies from our laboratory have used a small cohort of carefully diagnosed ME/CFS patients (the Dunedin cohort) and carried out extensive molecular studies utilizing the principles of precision medicine [33] with strict statistical limits. We have identified highly significant differences between the patient and control groups [10, 26].

The current research has extended these analyses by exploring the expressed proteins in peripheral blood mononuclear cells (PBMCs) from the Dunedin cohort, compared with their age- and gender- matched control subjects. It has utilized for the first time the comprehensive data independent acquisition method of SWATH-MS to identify affected biological pathways. Liquid chromatography coupled to tandem mass spectrometry (LC–MS/MS) has developed into the technology of choice for high-throughput characterization of proteins and proteomes [34], particularly in the initial discovery phase. SWATH-MS is a recently emerged LC–MS/MSALL strategy that allows systematic, unbiased detection and quantitation of detectable compounds in a sample. Individual proteins, or their patterns of expression or “biosignatures”, may yet prove to be valuable biomarkers in a diagnostic assay for ME/CFS and may also prove effective in gauging disease severity, dynamic variations in symptomatology, and longitudinal alterations assessing relapse and recovery phases of the illness, or other important factors like treatments [10, 34]. Given that at present, in some countries and among some health practitioners, there is still controversy over whether ME/CFS is a legitimate medical condition, proteome, transcriptome, and metabolome profiles can provide valuable initial objective evidence for the legitimacy of ME/CFS as a distinct disease.

This’discovery’ study was designed to cast a wide net to maximize the identification of as many proteins as possible in the study samples, as failure to identify them at this initial stage in the broad discovery list would preclude them from future examination for their involvement in biological pathways or their validation. The proteome analysis presented here, while further establishing the disturbance in regulation of immune and inflammatory biological pathways in ME/CFS, also provides evidence of histone methylation and proteasome activation. Most significantly, however, a large number of mitochondrial proteins were increased in expression, particularly those involved in the complexes of the electron transport chain, specifically Complex I (NADH Coenzyme Q oxidoreductase), oxidative phosphorylation (OXPHOS) (complex 5), the oxidative stress response and the TCA cycle. These changes in key proteins involved in the complexes of energy production and regulation of reactive oxygen species (ROS) form the main focus for this paper, as a novel and significant result of the analysis. The results suggest there is a deficiency in ATP production and mitochondrial function, compensated for by increased expression of proteins involved in the key ATP generating pathways. It complements a recent comprehensive study that also found increased in expression of the OXPHOS proteins, coupled with deficient ATP production by Complex V (ATP synthase) leading to their hypothesis of ME/CFS patients having a deficit in spare respiratory capacity in times of physical or other stress [27].

## Materials and methods

### Peripheral blood mononuclear cell (PBMC) collection and isolation

The study had approvals from by Health and Disability Ethics Committees in New Zealand (NTY/12/06/055 and 15,426). Eleven ME/CFS patients diagnosed according to the 2003 Canadian consensus criteria (CCC) [35] by an experienced ME/CFS clinician, were recruited along with age- and gender-matched controls (Table 1). All participants gave their informed signed consent to participate. The control group had no history of significant illness, injury, or fatigue related disorders. ME/CFS patients completed a detailed questionnaire, developed in-house, providing their clinical history and current health status.

**Table 1:** The ME/CFS study cohort

Clinical characteristics	ME/CFS participants	Control participants
Number	11	9
Median age (years)	43.2	38.0
Age range (years)	11.3-69	12.5-60
Gender	F = 7	F = 6
	M = 4	M = 3
Median BMI	23.7	^b^
Nationality	NZ/European	NZ/European
Median illness duration (years)	11	N/A
Stage of illness^a^	Acute = 2 Chronic = 9	N/A
Potential initial ME/CFS ‘trigger’	Acute infection = 7 Surgery = 1 Stress = 1 Unsure = 2	N/A

^a^self-reported

^b^Data not collected

Within 4 h of collection, whole blood samples were diluted 1:1 with filter-sterilized PBS, layered onto Ficoll-Paque PLUS (Sigma-Aldrich), and centrifuged at 400×*g* for 40 min. The PBMC interface layer was removed and washed twice with filter-sterilized PBS. The isolated PBMCs were then mixed with RNAlater (Thermo Fisher Scientific) (1:5 ratio) for storage.

### Cell lysis and protein digestion

Frozen pellets of isolated PBMCs were reconstituted in 200 µl of lysis buffer containing 0.2% (m/v) SDS (sodium dodecyl sulfate), 1 mM EDTA (ethylenediaminetetraacetic acid), 1 mM EGTA (ethylene glycol-bis(β-aminoethyl ether)-N,N,N′,N′-tetraacetic acid), 5 mM TCEP (tris(2-carboxyethyl)phosphine) in 40 mM Tris-base (tris(hydroxymethyl)aminomethane). The homogenates were sonicated for 2 min and then centrifuged at 16,000x*g* and 20 °C for 30 min. The supernatants were removed and diluted with 200 µl of detergent depletion buffer (8 M urea in 100 mM tri-ethyl ammonium bicarbonate (TEAB)). All samples were then further processed by the filter-aided sample processing (FASP) protocol [36]. Protein was then digested on filters with sequencing grade trypsin (Promega) using a trypsin/protein ratio of 1/20. After an overnight digestion samples were boosted with an additional aliquot of trypsin and further digested for 4 h. The digestion was then stopped by acidification with formic acid at a final concentration of 0.1%. One-third of each sample was pooled into a single sample for peptide fractionation by off gel-isoelectric focusing (OG-IEF). The remainder of the individual samples and the pooled sample were dried using a centrifugal vacuum concentrator.

### Peptide fractionation and shotgun proteomics

To identify the proteome of PBMCs from the patient and control cohorts and build a comprehensive spectral library the pooled samples aliquot was digested with trypsin, and the peptides subjected to fractionation by OG-IEF. Peptides were first purified by solid phase extraction on Sep-Pak Plus Light C18 cartridges (Waters) and then fractionated into 12 fractions by OG-IEF along a linear pH gradient from 4 to 10 using a 3000 OFFGEL fractionator (Agilent) according to the manufacturer’s protocol. Each fraction was then analysed in duplicates by data-dependent acquisition mass spectrometry using a 5600 + Triple Time-Of-Flight (TOF) mass spectrometer coupled to an Eksigent “ekspert nanoLC 415″ uHPLC system (AB Sciex). Therefore, peptides were separated on a 75 µm ID silica emitter tip column that was in-house packed with Luna (Phenomenex) C18 bead material (3.2 µm, 100Å) on a length of 20 cm. The LC gradient between mobile phase A (0.1% formic acid in water) and mobile phase B (0.1% formic acid in 90% aqueous acetonitrile) was developed in four gradient steps at a flow rate of 400 nL/min: (1) metered sample injection through a 5 µl loop followed by 3 min equilibration at constant 5% phase B; (2) linear increase of phase B to 25% over 90 min.; (3) linear increase of phase B to 40% over 20 min.; (4) linear increase of phase B to 95% over 10 min. The column was then washed at 95% phase B for 1 min and re-equilibration in 5% phase B for 5 min. The mass spectrometer was operated in data-dependent acquisition mode using the following instrument settings: The precursor ion measurement in the mass range of 400−1300 m/z was followed by collision-induced dissociation (CID) fragment ion measurements at rolling collision energy of the top 20 and top 30 precursors ions per cycle for the first and second technical replicate respectively. Three repeat measurements of each precursor were allowed during a period of 120 s. The ion accumulation time for the precursor and product ion scans were 250 and 120 ms respectively.

### Swath-ms

For protein quantification, each individual sample was analysed in technical triplicates by Sequential Windows Acquisition of all Theoretical Fragment Ion Spectra-mass spectrometry (SWATH-MS) using the same instrumentation and LC-gradient as described for the data-dependent acquisition (DDA) analysis. For SWATH-MS the mass spectrometer was operated in data-independent acquisition (DIA) mode performing a precursor ion scan in the mass range of 400−1300 m/z with an ion accumulation time of 50 ms followed by the acquisition of 33 consecutive fragment ion spectra from variable m/z isolation window sizes. The window sizes were calculated based on the precursor ion densities within the different m/z regions of a representative DDA analysis using the SWATH Variable Window Calculator application (AB Sciex). The ion accumulation time for each fragment ion spectrum was 100 ms in high sensitivity mode, which results in a total cycle time of about 3.4 s. Collision energy per window was set using automated rolling collision energy with a spread of 5 V.

### Data analysis

For protein identification and building of a spectral library raw data were searched against the human reference sequence database (comprising 87,570 sequence entries, downloaded from the NCBI server (https://www.ncbi.nlm.nih.gov/) on 29/03/2019) using the ProteinPilot software version 4.5 (AB SCIEX). The following search parameters were configured: the cleavage enzyme was trypsin, biological modifications and single amino acid exchanges were allowed. Peptide identification at a false discovery rate (FDR) of ≤ 1% and a confidence of ≥ 95% were accepted as significant and loaded into the SWATH Acquisiton MicroApp 2.0 integrated into the PeakView software version 2.2 (AbSciex) to build a spectral library. The spectral information from the individual DIA raw data was then aligned to the library spectra using a time window of 12 min. and a mass accuracy of 50 ppm for peak matching. The peak intensities of the 6 strongest fragment ions from each of the 10 strongest peptides per protein were then extracted from each DIA run where the threshold values of FDR ≤ 1% for matching peaks to the library spectra and confidence ≥ 99% for peptide identification were met in at least one sample. The intensity values were then imported into the MarkerView software version 1.2 (AB Sciex) for quantification. Global normalisation based on the total sum of peak intensities, unsupervised multivariate statistical analysis using principal component analysis (PCA) and Student’s *t* test was performed in the MarkerView software for sample grouping and comparison. Initially a t-test of the averaged technical replicates was carried out comparing the ME/CFS group with the healthy control group, generating a dataset of proteins with significantly different relative abundances between the two groups (*P *< 0.01, log_10_(fold-change) > 0.2 and < −0.2). These proteins were investigated using STRING (http://string-db.org, version 11) to provide information on functional association networks and identify potential interactions, and the Database for Annotation, Visualization and Integrated Discovery (DAVID) to further elucidate the function of the target proteins and their potential role in disease pathogenesis.

A PCA of the averaged technical replicates of all ME/CFS patient versus control samples, with 2970 proteins included in the quantification (having at least one peptide at a FDR ≤ 1% for matching the peaks in the spectral library) separated the samples based on expression patterns identified in principal components 1 (30.5%) and 2 (17.5%) into two groups. One group, termed ‘ME/CFS’, included nine of the eleven ME/CFS samples that showed similar PC2 clustering. The ‘control’ group included all control subjects. Two ME/CFS subjects (P10 and P11) that clustered closer with the control subjects were excluded from the following analysis. Selected proteins after a t-test comparison of the ‘ME/CFS’ and ‘control’ groups (*P *< 0.01 and a Log_10_(Fold-Change) > 0.2 and < −0.2) were investigated for stringent biological analysis. STRING database software generates a confidence score for each protein’s mutual information, based on available evidence. The nearer the score is to 1, the greater the confidence that there is interaction between the target proteins, (1 being the highest). We selected a confidence score > 0.7 to submit to DAVID and build protein–protein interaction (PPI) and functional association networks. The GO functions including biological processes, molecular functions and cellular components, along with KEGG and Reactome pathways, and PFAM, INTERPRO and SMART protein domains enriched by the PPI networks were described for all analyses.

DAVID was used for functional annotation clustering of the protein interactions and functions. Functional annotation clusters groups and displays similar annotations together, which makes the biology clearer and more focused. The grouping algorithm used is based on the hypothesis that similar annotations should have similar gene members. The group enrichment score is the geometric mean (in -log scale) of the member’s p-values (Fisher Exact/EASE score) in a corresponding annotation cluster and is used to rank their biological significance. Thus, the top ranked annotation groups most likely have consistent lower p-values for their annotation members.

## Results

### SWATH-MS and principal component analysis

**Fig. 1:**
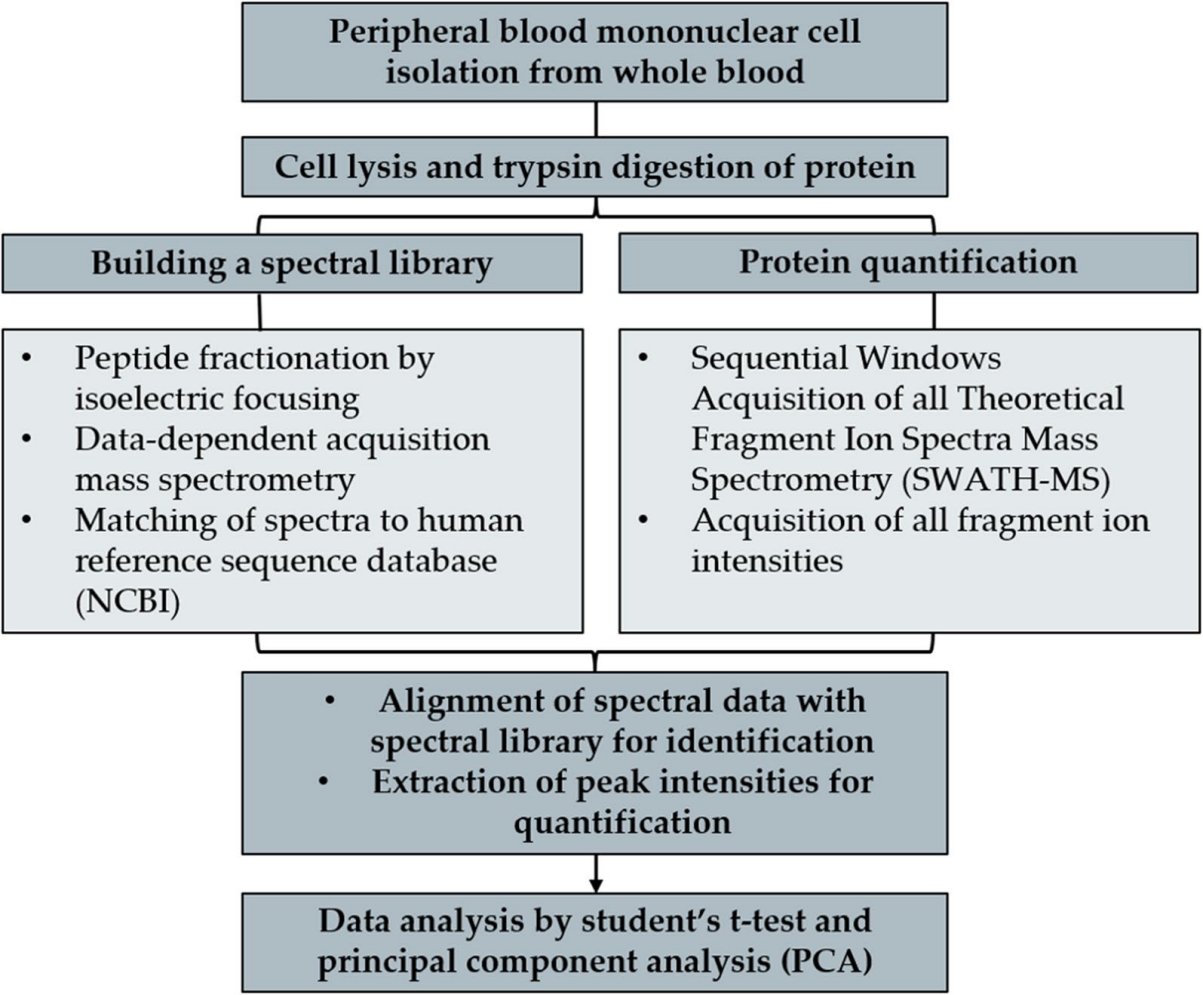
Schematic diagram of the SWATH-MS experimental protocol

The strategy of our SWATH analysis is shown in Fig. 1. Briefly, proteins were extracted from isolated PBMC and digested with trypsin. A third from each sample was added to a pooled sample, which was then subjected to peptide pre-fractionation using OG-IEF and analyzed by data-dependent acquisition MS to build a spectral library of 3990 proteins identified at a FDR ≤ 1% and a peptide confidence of ≥ 95%. The remainder of each individual sample was analyzed by SWATH-MS. The fragment ion intensities measured in the SWATH-MS analysis were aligned to the spectral library for identification and quantification. A total of 2970 proteins were quantified from triplicate sample measurements.

At the outset, a t-test comparison of the patient group vs the control group was carried out and proteins with significantly different relative abundances of *P* < 0.01 and Log_10_(Fold-Change) > 0.2 and < −0.2 identified (Tables 2 and 3). A total of 60 proteins were identified with different relative abundances between the eleven ME/CFS subjects and nine matched controls, with 38 showing increased relative abundance and 22 decreased relative abundance (Table 2 (increased) and Table 3 (decreased)).

**Table 2:** Proteins with increased relative abundance in ME/CFS compared to healthy controls (*P *< 0.01, Log_10_(Fold-Change) > 0.2)

GI accession	Protein name	Gene name	*P*- value	log_10_ (fold-Change)	Fold-change
148727341	Serine-threonine kinase receptor-associated protein	STRAP	0.00019	0.21	1.61
22538467	Proteasome subunit beta type-4	PSMB4	0.00021	0.25	1.77
24371248	FUN14 domain-containing protein 2	FUNDC2	0.00048	0.25	1.80
41281564	WD repeat-containing protein 37	WDR37	0.00049	0.23	1.69
145275202	Isoaspartyl peptidase/l-asparaginase	ASRGL1	0.00050	0.21	1.61
148539872	Acetyl-CoA acetyltransferase, cytosolic	ACAT2	0.00072	0.21	1.62
7705558	Inositol-3-phosphate synthase 1	ISYNA1	0.0011	0.24	1.75
7019419	Nucleolar GTP-binding protein 2	GNL2	0.0013	0.38	2.38
4503165	cullin-3	CUL3	0.0014	0.45	2.80
44680136	d-beta-hydroxybutyrate dehydrogenase, mitochondrial	BDH1	0.0014	0.41	2.60
4502205	ADP-ribosylation factor 4	ARF4	0.0019	0.27	1.86
259155315	Mitochondrial 2-oxoglutarate/malate carrier protein	SLC25A11	0.0022	0.24	1.73
13129110	Methylosome protein 50	WDR77	0.0023	0.24	1.75
4506923	SH2 domain-containing protein 1A	SH2D1A	0.0023	0.28	1.90
6912388	Grancalcin	GCA	0.0024	0.25	1.79
115270970	Chloride channel CLIC-like protein 1	CLCC1	0.0024	0.24	1.74
5031981	26S proteasome non-ATPase regulatory subunit 14	PSMD14	0.0029	0.32	2.09
188219591	Nucleolysin TIA-1 isoform p40	TIA1	0.0030	0.27	1.85
22035672	Thioredoxin reductase 2, mitochondrial	TXNRD2	0.0031	0.34	2.18
5454166	Vesicle transport through interaction with t-SNAREs homolog 1B	VTI1B	0.0033	0.29	1.93
7657116	Glyceraldehyde-3-phosphate dehydrogenase, testis-specific	GAPDHS	0.0033	0.35	2.23
225543288	SUMO-activating enzyme subunit	SAE1	0.0035	0.25	1.80
47132595	Phosphate carrier protein, mitochondrial	SLC25A3	0.0036	0.32	2.09
28373194	Proteasomal ubiquitin receptor ADRM1	ADRM1	0.0040	0.43	2.69
153251272	Calcineurin-like phosphoesterase domain-containing protein 1	CPPED1	0.0041	0.21	1.61
4505023	Proteasome assembly chaperone 1	PSMG1	0.0050	0.49	3.09
300360515	Actin-related protein 2/3 complex subunit 1A	ARPC1A	0.0051	0.23	1.71
183396804	Regulation of nuclear pre-mRNA domain-containing protein 2	RPRD2	0.0056	0.35	2.26
4885375	Histone H1.2	H1-2	0.0058	0.25	1.79
48762926	Periodic tryptophan protein 2 homolog	PWP2	0.0064	0.36	2.30
4885373	Histone H1.1	H1-1	0.0065	0.29	1.95
4885379	Histone H1.4	H1-4	0.0068	0.24	1.74
5032087	Splicing factor 3A subunit 1 isoform 1	SF3A1	0.0069	0.20	1.59
15487670	Nuclear RNA export factor 1 isoform 1	NXF1	0.0073	0.21	1.61
4885377	Histone H1.3	H1-3	0.0073	0.26	1.83
330340389	Up-regulated during skeletal muscle growth protein 5	USMG5	0.0088	0.23	1.70
4506741	40S ribosomal protein S7	RPS7	0.0090	0.23	1.69
4885381	Histone H1.5	H1-5	0.0095	0.29	1.94

**Table 3:** Proteins with decreased relative abundance in ME/CFS compared to healthy controls (*P *< 0.01, Log_10_(Fold Change) < -0.2)

GI accession	Protein name	Gene name	*P*-value	log_10_ (Fold change)	Fold change
7706495	dnaJ homolog subfamily B member 11	DNAJB11	0.00032	−0.24	0.57
4557367	Bleomycin hydrolase	BLMH	0.00045	−0.36	0.43
34101286	Zinc finger RNA-binding protein	ZFR	0.00047	−0.28	0.52
14149916	src-like-adapter 2	SLA2	0.00054	−0.23	0.58
66933005	Calnexin	CANX	0.0012	−0.24	0.58
6715607	Hemoglobin subunit gamma-2	HBG2	0.0014	−0.51	0.31
5031977	Nicotinamide phosphoribosyltransferase	NAMPT	0.0014	−0.31	0.49
8923541	UPF0587 protein C1orf123	C1orf123	0.0017	−0.46	0.35
10863927	Peptidyl-prolyl cis–trans isomerase A	PPIA	0.0026	−0.26	0.54
143770741	Platelet glycoprotein VI	GP6	0.0028	−0.21	0.61
19913385	Protein G6b	C6orf25	0.0038	−0.42	0.38
4506085	Mitogen-activated protein kinase 13	MAPK13	0.0039	−0.27	0.54
4504073	Platelet glycoprotein Ib beta chain	GP1BB	0.0045	−0.26	0.55
12545406	ras GTPase-activating protein 1	RASA1	0.0053	−0.20	0.63
4757774	ADP-ribosylation factor-like protein 3	ARL3	0.0067	−0.24	0.58
410173533	PREDICTED: uncharacterized protein LOC100996504	ENSG000 263264	0.0070	−0.27	0.54
29826335	Eukaryotic translation initiation factor 2 subunit 2	EIF2S2	0.0080	−0.25	0.56
30181236	copine-2	CPNE2	0.0087	−0.31	0.49
190014603	TBC1 domain family member 13	TBC1D13	0.0087	−0.26	0.55
4504077	Platelet glycoprotein IX	GP9	0.0089	−0.31	0.49
157738645	Plexin-A4	PLXNA4	0.0092	−0.38	0.42
4504351	Hemoglobin subunit delta	HBD	0.0093	−0.33	0.47

The identified proteins were investigated by web-based applications STRING (http://string-db.org, version 11) and the Database for Annotation, Visualization and Integrated Discovery (DAVID) (https://david.ncifcrf.gov/, version 6.8). The increased abundance proteins have 32 functional associations (CI > 0.4) compared to an expected number of 13 random interactions, and a PPI enrichment *P* = 3.2 × 10^−6^ (see Additional file 1: Table S1). The decreased relative abundance proteins have 6 functional associations (CI > 0.4) compared to an expected number of 1 random interaction, and a PPI-enrichment *P* = 0.0016 (see Additional file 1: Table S1). Additional file 1: Table S2 and S3 show the biological processes, molecular functions and cellular components (GO annotations) involving the proteins identified as changed in the ME/CFS group. KEGG and Reactome pathways, UniProt keywords and PFAM, INTERPRO and SMART protein domains implicated by the differently abundant proteins. In summary, the increased abundance proteins in ME/CFS function in histone methylation and DNA Damage/Telomere Stress Induced Senescence, proteasome assembly, NAD, NAD(P)-binding and mitochondrial substrate/solute transport, while decreased abundance proteins were primarily linked to wound healing, platelet activation and adhesion, blood coagulation and oxygen transport roles.

**Fig. 2:**
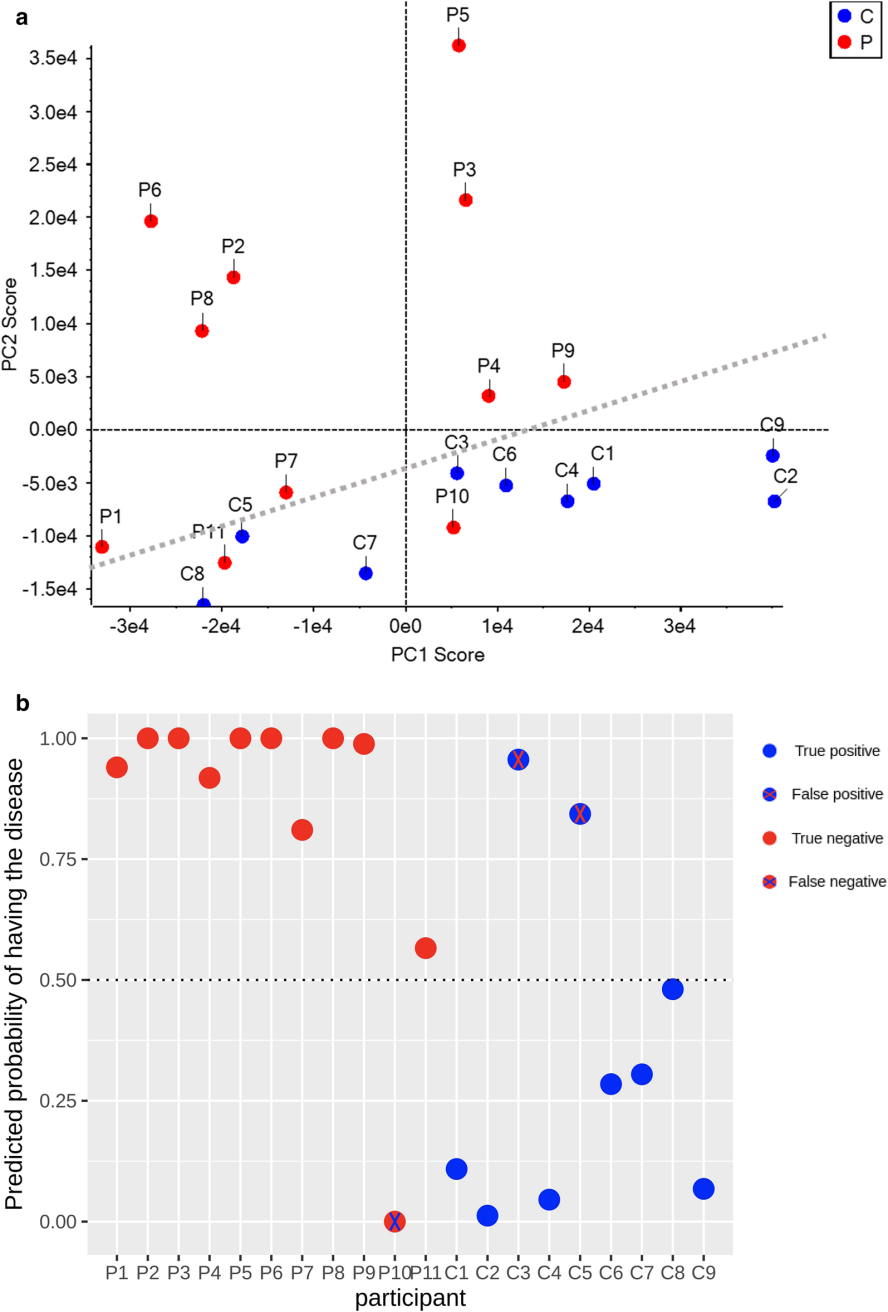
**a** Principal component analysis of the study group proteomic data. Scores for PC1 (38.5%) versus PC2 (15.9%), Sqrt/Pareto. The nine age- and gender-matched healthy control subjects grouped close together, distinct from a large subgroup of nine (P1, P2, P3, P4, P5, P6, P7, P8, and P9) of the eleven ME/CFS subjects. **b** Principal Component logistic regression after leave-one-out cross-validation classified 10 out of 11 patients correctly as patients and 7 out of 9 controls correctly as controls. Patient P10 and controls C3 and C5 were misclassified

As the analysis was exploratory and, as has been shown in numerous studies of ME/CFS patient cohorts, it was likely there would be subgroups within the cohort, a principal component analysis (PCA) was used to further investigate the proteomes of the patient and control groups and to inform groupings. The identified principal components accounted for as much of the variability of the data as possible (PC1 38.5% of the data and PC2 15.9%), while having an orthogonal relationship. The PCA showed two possible subgroupings of study samples with similar proteome expression patterns, based primarily on their PC2 scores; one subgroup included nine ME/CFS patients (P1, P2, P3, P4, P5, P6, P7, P8, and P9), now called the ‘ME/CFS’ subgroup, while the second subgroup included all nine control participants and two ME/CFS subjects (P10 and P11) (Fig. 2).

The two different ME/CFS subgroups and control PCA clusters observed could not be explained by age, gender or BMI differences. Patients in our study might be separating by the differing severity of their symptoms, resulting in subtly different disease biology [9, 37]. Until our understanding of ME/CFS is more complete and a molecular diagnostic test is available, it is difficult to resolve better the concept of subgroups of ME/CFS.

To assess the ability of the principal components to predict patient or control, we fitted a logistic regression to the first three principal components. We then applied leave-one-out cross-validation. We found the model predicted the disease well, with 3/20 prediction errors (two false positives and one false negative). Figure 2B shows the predicted probabilities for all 20 subjects. We see that only P10, C3 and C5 were misclassified, on the expectation from the results of the PCA (Fig. 2a). A permutation test shows that the probability of achieving a prediction error of 3/20, or more extreme, is 0.04, so it is unlikely that the observed predictive capacity of the principal components has been caused by chance.

The nine ME/CFS patients that had separated from the controls were then separately compared with the nine controls. A student’s t-test between the two groups found now an expanded number of 99 proteins with significantly different abundance levels between this ‘ME/CFS’ group and ‘control’ group (P < 0.01, log_10_ (Fold-Change) > 0.2 and < −0.2, increased proteins n = 47; decreased proteins n = 52). A number of identified proteins were involved in mitochondrial functioning, the TCA cycle, oxidative phosphorylation and redox signaling (see below in Table 4).

**Table 4:** Differently abundant mitochondria-related proteins in the ‘ME/CFS’ PCA group compared to the ‘control’ group (P < 0.01, log_10_(Fold-Change) > 0.2) but also including proteins (shaded) with P < 0.05 and Fold-Change > 1.3 and < 0.75

Increased relative abundance proteins	P-value	Fold-change
FUN14 domain-containing protein 2	0.00023	1.91
NADH dehydrogenase [ubiquinone] 1 beta subcomplex subunit 3	0.00034	1.60
d-beta-hydroxybutyrate dehydrogenase	0.00090	2.89
Inositol-3-phosphate synthase 1	0.0025	1.82
Mitochondrial 2-oxoglutarate/malate carrier protein	0.0042	1.78
Phosphate carrier protein	0.0060	2.22
ATP synthase subunit epsilon	0.0079	1.70
Glyceraldehyde-3-phosphate dehydrogenase, testis-specific	0.0081	2.31
Thioredoxin domain-containing protein 17	0.0065	1.58
Prohibitin	0.0079	1.40
Selenoprotein H	0.013	1.62
Endophilin-B1	0.014	1.46
Thioredoxin reductase 2	0.014	2.18
Up-regulated during skeletal muscle growth protein 5	0.014	1.79
Glutamine-dependent NAD(+) synthetase	0.014	1.53
Poly [ADP-ribose] polymerase 4	0.015	1.65
NADH dehydrogenase [ubiquinone] 1 alpha subcomplex subunit 3	0.015	1.58
NADH dehydrogenase [ubiquinone] 1 beta subcomplex subunit 11	0.016	1.64
ATP synthase subunit delta	0.017	1.78
Poly [ADP-ribose] polymerase 14	0.018	1.78
Single-stranded DNA-binding protein	0.019	1.51
Ubiquinone biosynthesis protein COQ9	0.020	1.77
NADH dehydrogenase [ubiquinone] 1 alpha subcomplex subunit 12	0.020	1.76
Short-chain specific acyl-CoA dehydrogenase	0.020	1.49
Glycerol-3-phosphate dehydrogenase	0.021	1.40
2-oxoglutarate dehydrogenase	0.025	1.35
Aconitate hydratase	0.025	1.36
39S ribosomal protein L43	0.027	1.55
Cytochrome c oxidase assembly factor 6 homolog	0.028	1.47
Biogenesis of lysosome-related organelles complex 1 subunit 1	0.030	2.04
Pyruvate dehydrogenase E1 component subunit beta	0.032	1.31
BRI3-binding protein	0.033	2.27
Trifunctional enzyme subunit beta	0.034	1.40
ATP synthase subunit b	0.035	1.49
Putative transferase CAF17	0.036	1.30
Glycine–tRNA ligase	0.037	1.47
39S ribosomal protein L12, mitochondrial	0.038	1.63
Peptidyl-prolyl cis–trans isomerase NIMA-interacting 4 isoform 1	0.040	1.46
Isocitrate dehydrogenase [NAD] subunit beta, mitochondrial isoform a precursor	0.042	2.17
Mitochondrial fission 1 protein	0.047	1.48

Decreased relative abundance proteins	P-value	Fold-Change
Isocitrate dehydrogenase [NADP], mitochondrial precursor	0.0001	0.59
Peptidyl-prolyl cis–trans isomerase F, mitochondrial precursor	0.0005	0.38
Peroxiredoxin-6	0.0081	0.66
Thiosulfate sulfurtransferase	0.0092	0.65
Choline transporter-like protein 1	0.011	0.36
39S ribosomal protein L23, mitochondrial	0.012	0.45
Mitochondrial import inner membrane translocase subunit Tim8 A isoform 1	0.012	0.47
Cytochrome c	0.013	0.56
ATP synthase mitochondrial F1 complex assembly factor 1 isoform 2 precursor	0.013	0.60
ATP-binding cassette sub-family B member 7, mitochondrial isoform 1	0.018	0.55
Dynamin-1-like protein isoform 1	0.019	0.68
Stimulator of interferon genes protein	0.036	0.61
Oxidation resistance protein 1 isoform 4	0.037	0.69
NADH dehydrogenase [ubiquinone] 1 alpha subcomplex subunit 5	0.040	0.60
Glycerol kinase isoform b	0.047	0.70
CCA tRNA nucleotidyltransferase 1, mitochondrial	0.048	0.57

**Fig. 3:**
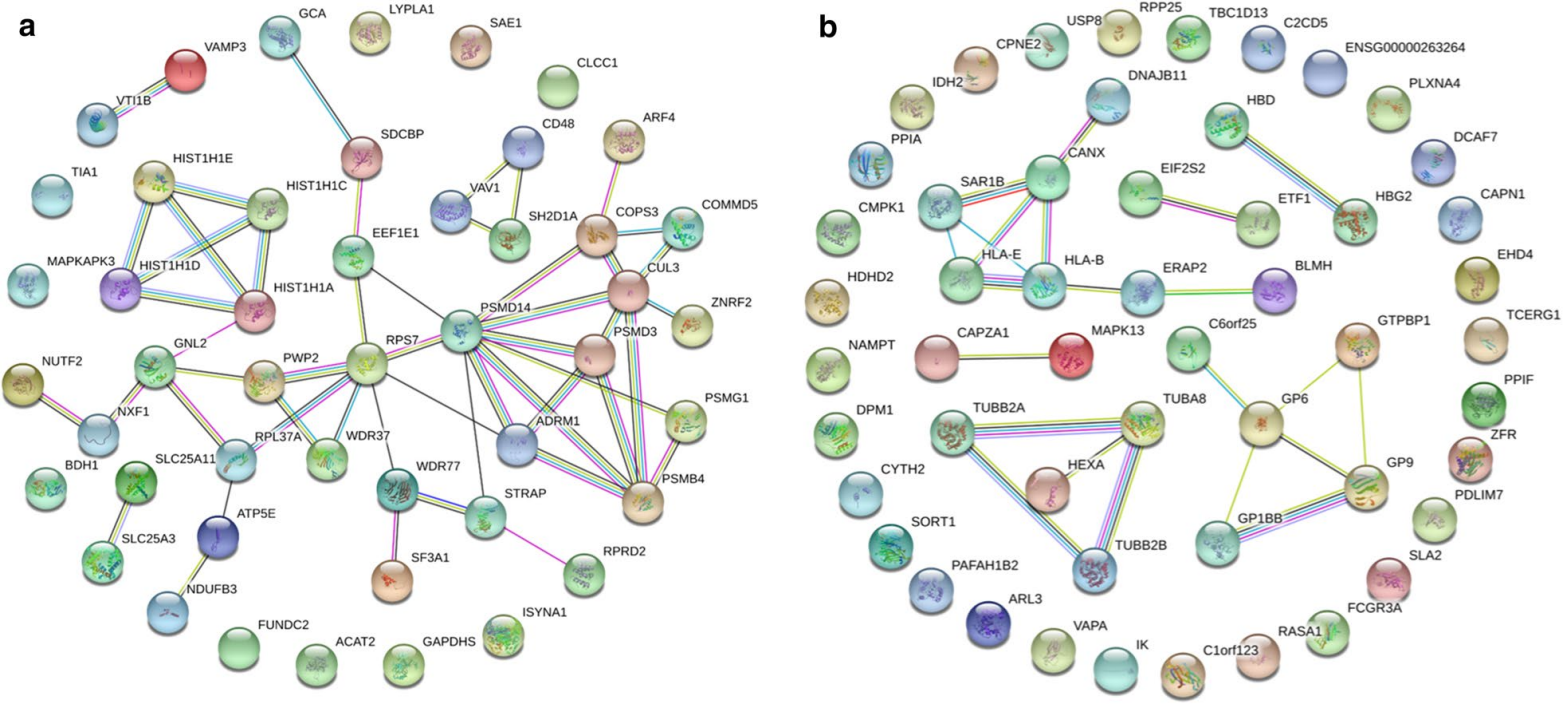
**a** STRING functional interaction networks of the 47 proteins increased in abundance in the PCA ‘ME/CFS’ group compared to ‘controls’ (*P* < 0.01, log_10_(Fold-Change) > 0.2) and in **b**. STRING functional interaction networks of the 52 proteins decreased in abundance in the PCA ‘ME/CFS’ group compared to ‘controls’ (*P* < 0.01, log_10_(Fold-Change) < −0.2)

STRING analysis of the proteins increased (Fig. 3a**)** and decreased (Fig. 3b in the ME/CFS subgroup complemented the findings from the initial full ME/CFS set. Overall, the functional associations of the differently abundant proteins in the subgroup of ME/CFS patients increased significantly, and the majority of the functional pathways identified when comparing all eleven ME/CFS participants with the control group were further supported and augmented by comparison with this ME/CFS PCA subgroup. Of these, the majority were involved in stress-induced senescence and oxidative stress, mitochondrial functional pathways, immune and inflammatory pathways, and proteasome activation. Additional proteasome-related proteins and mitochondrial proteins involved in the electron transport chain and ATP synthesis emerged as being further enriched. The decreased protein data set was expanded to include MHC class I immune proteins and ribosomal proteins. The 47 increased proteins had 50 functional interactions (CI > 0.4) compared to the expected 23 random interactions, with a PPI enrichment *P *= 4.28 × 10^−7^. Similarly, the 52 decreased proteins (with one protein excluded as the sequence mapped to no known protein/gene) had 22 functional interactions (CI > 0.4) compared to 11 expected random interactions, with a PPI enrichment *P *= 0.0015.

The identified mitochondrial proteins listed in Table 4 includes both increased and decreased relative abundance mitochondrial proteins with a lower P < 0.05 and Fold-Changes > 1.3 and < 0.75 (shaded). With these relatively less stringent P-value and Fold-Change cut-offs, there were a greater number of citric acid (TCA) cycle proteins and regulation of reactive oxygen species (ROS) proteins identified in the ME/CFS PCA group. We also tested the hypothesis that all identified mitochondrial proteins present among the 2970 proteins under investigation could alone specifically predict the disease with the above regression model (see Fig. 2b). Simulation showed that these 147 mitochondrion proteins were no better on average than a random sample of 147 other proteins.

Protein data sets were submitted to DAVID for functional annotation clustering. Analysis included (i) the significantly increased and decreased (*P* < 0.01, log_10_(Fold-Change) > 0.2 and < −0.2) proteins identified from comparison of all eleven ME/CFS participants and the nine matched controls, and (ii) the second protein dataset generated from the PCA selected subgroup of nine ME/CFS subjects compared with controls, all with medium confidence (CI > 0.4 combined score from String functional association analysis) or higher interactions. Even with the small number of proteins identified with different relative abundance in the full set of ME/CFS subjects in comparison to controls (increased n = 38 and decreased n = 22, see Tables 2 and 3 respectively), interesting biological pathways were either enriched (6 functional clusters) or depleted (2 functional clusters) in the ME/CFS group (see Additional file 1: Figures S1 and S2—increased relative abundance proteins, and S3–decreased relative abundance proteins). Supporting the STRING analysis, histone methylation and regulation of gene expression was indicated to be an enriched biological implication of the increased protein data set. Also noted, WD repeat containing proteins were enriched, along with NAD and NAD(P)-binding Rossman-like fold proteins, proteins involved in proteasome poly-ubiquination of proteins and the MAPK cascade, inner membrane proteins of the mitochondrion, and proteins of the Golgi apparatus. Proteins decreased in the ME/CFS group were involved in blood coagulation and platelet activation and protein folding.

The network of proteins with medium or higher confidence interactions (CI > 0.4 combined score, Additional file 1: Table  S1) of differentially abundant proteins (n = 99, *P* < 0.01, log_10_(Fold-Change) > 0.2 and < –0.2) after t-test comparison of the ME/CFS and control PCA groups were also analysed by DAVID functional association clustering. The 47 increased proteins clustered into several key categories, specifically; histone DNA binding and nucleosome assembly (enrichment score = 3.25); proteasome function, polyubiquination and the TNF-mediated signalling pathway (enrichment score = 2.16), WD repeat domains (enrichment score = 1.59) the mitochondrion and inner mitochondrion membrane (enrichment score = 1.24), and NAD and NAD(P)-binding domains (enrichment score = 1.22). The 52 decreased proteins also clustered into functional groups including antigen processing and presentation (enrichment score = 2.37); tubulin and the cytoskeleton (enrichment score = 2.25); endoplasmic reticulum (enrichment score = 2.03); and cell–cell adhesion (enrichment score = 0.73).

## Discussion

### Evidence of mitochondrial dysfunction in this study

The most striking result from this proteome analysis was the number of different mitochondrial related proteins increased in expression in the ME/CFS group compared to controls, particularly after PCA grouping and analysis (Fig. 3a, and Table 4). Several of these mitochondrial proteins were also increased when the full set of eleven ME/CFS participants were compared to the matched control subjects (Table 2). These included D-beta-hydroxybutyrate dehydrogenase (BDH1) and acetyl-CoA acetyltransferase containing 2 (ACAT2), both playing roles in fatty acid metabolism and the synthesis and degradation of ketone bodies. Mitochondrial 2-oxoglutarate/malate carrier protein isoform 2 (SLC25A11), has multiple roles being involved in (i) transporting 2-oxoglutarate across the inner mitochondrial membrane and playing a role in gluconeogenesis from lactate, (ii) maintaining mitochondrial fusion and fission, and (iii) in the organization and morphology of the mitochondrial cristae [38], and phosphate carrier protein, mitochondrial isoform b (SLC25A3), transports phosphate into the mitochondria for oxidative phosphorylation. FUN14 domain containing 2 (FUNDC2), in the outer mitochondrial membrane, and thioredoxin reductase 2 (TXNRD2), involved in mitochondrial redox homeostasis were also identified. Together, these changes suggest that in the ME/CFS group mitochondrial activity may be elevated, perhaps to the point where harmful levels of reactive oxygen species (ROS) are reached and sustained.

The interesting functional roles of these proteins became clearer with the expanded protein dataset arising from the comparison of the subgroup of ME/CFS subjects versus the control subjects based on the clustering in the PCA analysis where the study subjects segregated based on proteomic data, rather than simply on ME/CFS or control designations. Those ME/CFS subjects clearly differing from their matched controls exhibited an increased number of mitochondrial proteins with changed relative abundance (see Table 4). Most significantly, Complex I proteins, TCA cycle proteins, redox regulation proteins Complex III proteins and Complex V (ATP Synthase) proteins were present in significantly different (and primarily increased) amounts in ‘ME/CFS’ compared to ‘controls’. The results suggest that this majority subgroup of ME/CFS patients have dysregulation of mitochondrial energy production components, in particular seen by significant increases in several key Complex I proteins.

**Fig. 4:**
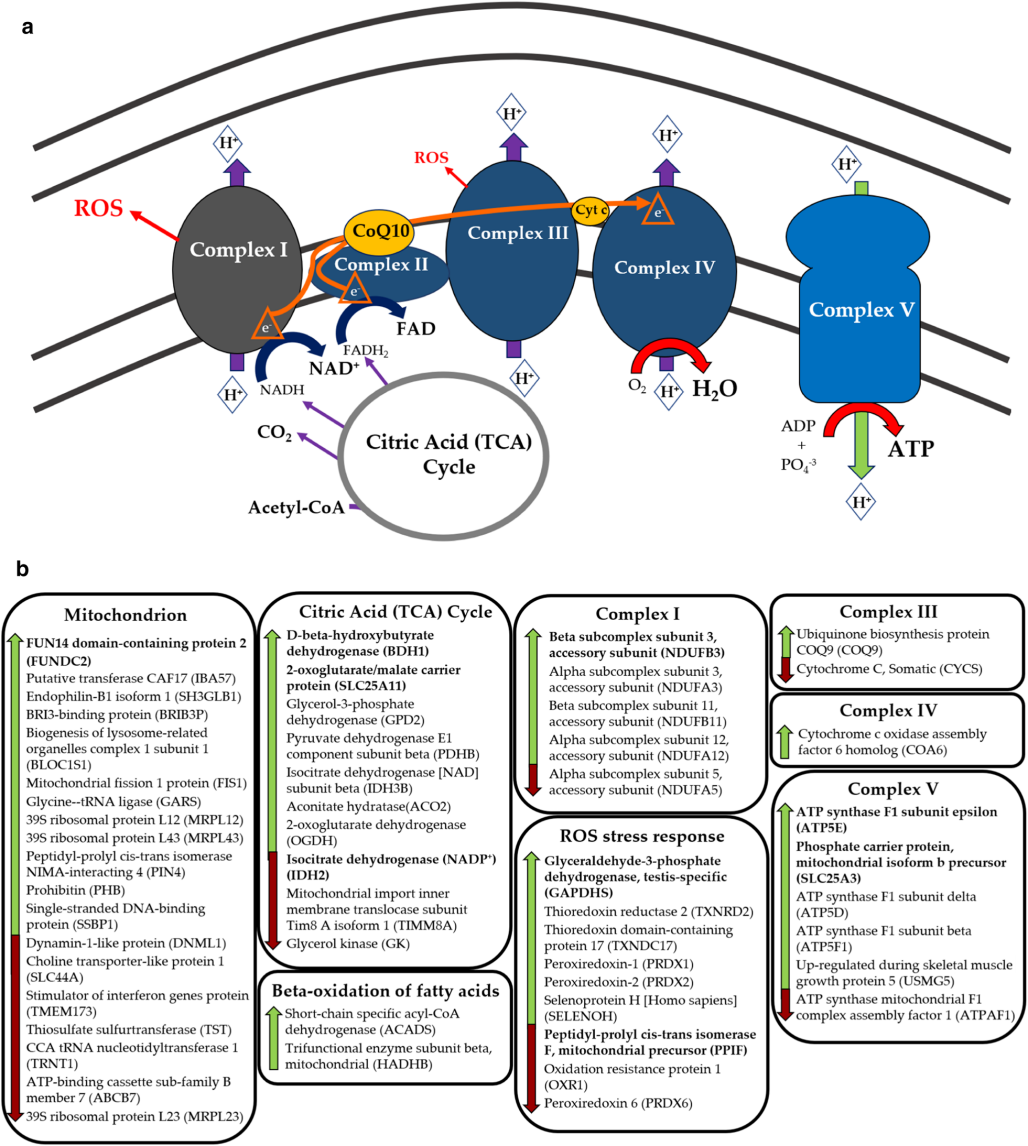
**a** A schematic of the mitochondrial respiratory chain constructed by the authors to highlight the OXPHOS complexes, the major reactive oxygen species (ROS) production sites, and the ATP Synthase complex relevant to the differential expression of mitochondria-related proteins in the ME/CFS group **b** Highlighting differentially abundant proteins (P < 0.05, log_10_(Fold Change) > 0.114 and < −0.125) involved in mitochondrial function and energy metabolism. In bold are proteins with P < 0.01 and log_10_(Fold Change) > 0.2 and < −0.2. The green arrow represents increased relative abundance and the red arrow decreased relative abundance in the ‘ME/CFS’ PCA group, compared to controls

Many of the identified mitochondrial proteins increased in expression (see Fig. 4) were involved in proper functioning of OXPHOS complex in the inner membrane of the mitochondrion. Of the OXPHOS complexes, Complex I and Complex V (ATP Synthase), had a significant number of their constituent proteins with increased expression compared to controls (NDUFA3, NDUFB3, NDUB11 and NDUB12 in Complex I, and ATP5E, ATP5D, ATP5F1 and USMG5 in Complex V). Enhanced ATP metabolic processes, generation of precursor metabolites and energy, oxidative phosphorylation, redox homeostasis and increased recycling of NAD, were identified by both STRING and DAVID functional association analysis in the ME/CFS PCA cohort. While primarily increased abundance of mitochondrion-related proteins was seen in the ME/CFS PCA group (Fig. 4), there were several important proteins with decreased relative abundance. These included PRDX6 (whole cell body) and OXR1with roles in redox regulation, and some proteins involved in the assembly of the mitochondrion such as TIMM8A. Mitochondrial IDH2, also relatively decreased in the ME/CFS PCA subgroup, uses NADP(+) as an electron receptor to catalyze its forward oxidative decarboxylation reaction in cellular defense against oxidative damage [39].

The findings suggest that in ME/CFS there are raised levels of Complex I and Complex V constituents, and increased efforts to enhance ATP production. As Complex I is the major producer of ROS in the electron transport chain, the identification of apparently dysregulated redox homeostasis in ME/CFS is important to note. Dysregulated abundance of important redox regulatory proteins implies a response to increased ROS in the cells, as well as the potential inability to cope with high levels of ROS produced in the mitochondrion. The results of this study suggest that in future studies, consideration of ATP levels and ROS production in biological samples will be important. Unravelling mitochondrial function by Seahorse analysis (i.e. measuring oxygen consumption and extracellular acidification to determine the efficiency of different mitochondrial parameters, for example ATP production and maximal uncoupled respiration) would also be of interest.

### Histone methylation and proteasome activation; dampened platelet activation and immune processes

Although differentially expressed mitochondria-related proteins evolved from the study to be the main interest and focus of discussion, it is worth noting that significant changes were also observed between the ME/CFS cohort and control cohort in proteins involved in histone methylation, immune inflammation and proteasome activation functional networks (Table 2 and 3 and Additional file 1: Tables S2, S3 and S4). Histone methylation pathways highlighted by both STRING and DAVID analysis indicated histone methylation at H3K4 (activating nearby gene expression) and H3K27 (repression of gene expression). Differential methylation patterns have been observed in ME/CFS by us [40] and other research groups, many in immune-related genes [41]. Epigenetic modifications have been suggested to play important roles in inflammatory and autoimmune diseases, diseases which share many similarities with ME/CFS [20]. Multiple DNA methylation studies have now shown both hypo-methylation and hyper-methylation at specific gene promoters in ME/CFS patients, including in our own ongoing patient/control study. In our study, hyper-methylation changes were proportionally much higher at promoters than across the whole genome, where loss of methylation accounted for most of the changes between the ME/CFS study group and the controls [10]. The addition of methyl groups at promoters is consistent with a hypometabolic state where the expression of genes involved in metabolic pathways is down regulated. Differential methylation in ME/CFS has been found in close proximity to genes involved in immune function and cellular metabolism, linked with the ME/CFS phenotype [21, 22, 42]. Overall, these findings align with recent ME/CFS findings pointing towards impairment in cellular energy production and immune dysfunction in the patient population. Several proteasome proteins were also increased in the proteomes of both the full set of ME/CFS subjects and the PCA selected ‘ME/CFS’ subgroup (Table 2 and Additional file 1: Table S4). This apparent upregulation of the proteasome may be a response to misfolded proteins, a phenomenon also implicated in STRING and DAVID functional assessments, due to concurrent ROS damage and oxidative stress in ME/CFS PBMCS.

The observation of a dampening effect on platelet activation, and MHC class I immune functions, in ME/CFS is also of interest. Only surface platelet membrane glycoproteins were identified, along with activating factors, suggesting some platelet proteins remained in the PBMC fraction via interaction with them, and in higher amounts in controls than in ME/CFS. Activated platelets have an anti-inflammatory effect by interacting directly with monocytes via activating-type Fc receptors for IgG to enhance IL-10 production and reduce TNFα secretion [43]. It is also possible ME/CFS patients may have reduced platelets overall, or suppressed immune activation, that contributed to their lower abundance of platelet proteins. MHC Class I-related proteins were decreased in the ‘ME/CFS’ group. The function of this class of protein is to display intracellular peptides, generated mainly from degradation of cytosolic proteins by the proteasome to cytotoxic T cells. Interestingly proteasome activity and protein degradation does appear to be enhanced in the ME/CFS, suggesting the dampening down of the MHC Class I functional pathways may be a response to prevent unnecessary activation of the immune response. A transcriptome study of the same ME/CFS and control study group found among genes significantly differently expressed in ME/CFS, the three most significant and increased were *IL8*, *NFKBIA* and *TNFAI3P*, all functionally related as responders to over-activation of inflammatory NF-KB. STRING (*P* < 0.01) and ingenuity pathway analysis (*P* < 0.05) of the biological pathways affected by these changed genes including immune/inflammatory pathways, cellular stress response and oxidative stress, circadian clock function, metabolism and mitochondrial function [26]. The correlating gene transcripts encoding the respiratory complexes, TCA cycle proteins and ATP Synthase subunits were not noted to be significantly changed in the ME/CFS transcriptome, so may be regulated by post translational mechanisms to increase their proteins, however similar biological pathways were apparently enriched in both studies. Thus, these findings support the already substantial published evidence for immune dysregulation playing an important role in ME/CFS pathogenesis [44].

### Cellular senescence in ME/CFS

An important finding was the cluster of five histone H1 linker proteins (H1-1, H1-2, H1-3, H1-4 and H1-5) all present in higher relative abundance in the whole ME/CFS group when compared to control subjects. Enrichment of these proteins indicates enhanced chromatin remodelling and DNA methylation in ME/CFS. String analysis of the proteins with significantly changed relative abundance (*P* < 0.01, log_10_(Fold-Change) > 0.2 and < −0.2) suggests that the increased expression of these H1 linker histones, along with proteasome and redox regulatory proteins, reveals an ongoing stress response in ME/CFS PBMCs, leading to DNA/telomere damage induced senescence. The process of DNA damage/telomere stress induced senescence culminates in the formation of senescence associated heterochromatin foci (SAHF), a process in which the five histone proteins are key players. Cellular senescence is defined as the irreversible loss of division potential in somatic cells. Senescence has an important role in vivo, on the one hand protecting organisms against a build-up of cells containing DNA damage and on the other hand contributing to age-dependent tissue dysfunction [45, 46]. At present, cellular senescence has been found in a variety of tissues and diseases, such as cancer (lung cancer, breast cancer, neuroblastoma, astrocytoma, colorectal cancer, etc.), fibrosis (idiopathic pulmonary fibrosis, renal fibrosis, etc.), chronic obstructive pulmonary disease COPD, and pulmonary hypertension [45, 46]. Increased cellular senescence is usually attributed to the aging process, however this is not the case in our ME/CFS group, with study participant ages ranging from 11 to 69 years. This may be explained by evidence of persistent oxidative stress and cell damage from ROS in the ME/CFS group. The increases in respiratory chain complex proteins seen in the ME/FS group, particularly in the nine ME/CFS subjects, indicate excessive ROS production in ME/CFS and an imbalance between the antioxidant and oxidative system in the PBMCs. Mitochondria are the main source of ROS in cells and the main target of oxidative damage, which in turn can reduce the efficiency of mitochondria and induce ROS production. Thus, premature senescence in ME/CFS is a likely result of increased oxidative stress resulting from escalated and dysregulated mitochondrial activity.

Senescent cells, albeit not proliferating, are metabolically and transcriptionally active, thereby capable of affecting their microenvironment, notably via the production of inflammatory mediators. These mediators maintain and propagate the senescence process to neighbouring cells, and then recruit immune cells for clearing senescent cells. Thus, the chronic dysregulation of the immune response pathways seen in ME/CFS may too be linked to the observed mitochondrial dysfunction, oxidative stress and subsequent triggering of premature senescence [45, 46]. Interestingly, a study on longevity in mice highlighted the importance of electron transport chain (ETC) proteins, particularly the matrix arm subunit of Complex I [47]. Young long-lived mice had decreased levels of these ETC proteins, which is associated with improved Complex I assembly, higher Complex I-linked state 3 oxygen consumption rates and decreased superoxide production. The opposite was true in old mice. The study found that disruption of Complex I assembly effectively reduces oxidative metabolism and gives rise to mitochondrial superoxide production, with the effect rescued by knockdown of prohibitin (PHB), a mitochondrial chaperone [47]. Prohibitin levels were increased in our ME/CFS PCA subgroup, suggesting this rescue effect is not occurring (Table 4 and Fig. 4).

### How this proteome analysis compares to other ME/CFS proteome analyses and mitochondrial dysfunction

Proteomic studies, although not by SWATH-MS analysis, in more recent years have implicated mitochondrial involvement in ME/CFS, but with conflicting evidence. One group found reduced mitochondria biogenesis, but not normalized respiratory chain enzyme activities in the muscle of ME/CFS sufferers [48]. Consistent with this study, the elevated expression of mitochondrial proteins in serum, platelets and lymphocytes have been found in some studies [27, 49]. Conversely in others, mitochondrial function appeared reduced in isolated neutrophil cells, [28, 49], as did respiration in ME/CFS PBMCs [15], while oxidative phosphorylation complexes appeared unaffected [30, 31].

However, studies recently implicate a significant role for mitochondrial dysfunction in ME/CFS, consistent with the finding of this SWATH-MS study. An important study was a translational case–control study of monozygotic twins discordant for ME/CFS by Ciregia et al. (2016) [28], who performed proteomics on platelet mitochondria. Of the 1007 proteins detected, 194 mitochondrial proteins were significantly different in ME/CFS. Among these, 41 had a fold change greater than 2.0 (34 upregulated and 7 downregulated). This study highlighted similar protein expression changes to those presented from our PBMC proteome study and important biological pathways inferred to be affected were also found to be dysregulated in our SWATH-MS proteome study of the Dunedin ME/CFS cohort. For example, in the Cirega et al. (2016) study pathway analysis highlighted the ‘metabolism of NADH’ as one of the most important biological functions involved in ME/CFS [28]. Our results implicate higher levels of NADH would be produced due to increased abundance in proteins in the TCA cycle, along with potentially increased oxidation of NADH by upregulated Complex I. The NAD +/NADH ratio has an important role in regulating the intracellular redox status and, therefore, represents the metabolic state. Some studies have suggested that NADH concentrations are by contrast significantly lower in ME/CFS patients compared with healthy controls [50, 51]. Higher levels of malate dehydrogenase and isocitrate dehydrogenase were observed in the case–control study above and proposed to be an adaptive response to deficiencies of NADH which, in turn, would play a critical role in mitochondrial ATP production. Integrating the results of this study and our own, a plausible *hypothesis* is that *there is metabolic dysfunction in ME/CFS resulting in insufficient energy production and triggering compensatory increases in key OXPHOS proteins to ameliorate this deficiency*.

A recent paper provides significant and substantial support for this hypothesis [27]. This paper investigated immortalized lymphoblasts from 51 ME/CFS patients, assessing parameters of mitochondrial function by Seahorse extracellular flux analysis, proteomics, and biochemical assays. The most striking finding from these experiments was the observation that the rate of ATP synthesis by Complex V, as a proportion of basal oxygen consumption, was significantly reduced in ME/CFS [27]. Concurrently, significant elevations were seen in Complex I oxygen consumption rate (OCR), maximum OCR, spare respiratory capacity, non-mitochondrial OCR and “proton leak” as a proportion of basal OCR [27]. Also seen was upregulated expression of the mitochondrial respiratory complexes, fatty acid transporters and enzymes of the ß-oxidation and TCA cycles [27]. Together these findings support an illness model whereby a defect in Complex V, and therefore deficiency in ATP production, is accompanied by a compensatory upregulation of mitochondrial respiratory capacity and respiratory complexes, membrane transporters, and proteins involved in fatty acid ß-oxidation [27]. These compensatory mechanisms homeostatically enable adequate ATP synthesis in resting cells, however they will be unable to respond sufficiently to acute energy demands.

#### The key role of mitochondrial Complex I

The observation in our study, in Missailidis et al. (2019) [27] and in Cirega et al. (2016) [28] of significant increases in expression of Complex I proteins is worthy of further discussion. These key studies, and the proteome results presented here, strongly indicate oxidative damage is occurring in ME/CFS. Complex I is composed of 44 different subunits, and its assembly requires at least 13 specific assembly factors [52]. Complex I modular assembly depends on a tightly coordinated series of steps, with the different functional modules proposed to assemble separately and associate together afterwards to form the final enzyme [53]. Furthermore, Complex I is known to form supercomplexes with Complex III and IV in the inner mitochondrial membrane, helping to form the cristae folds within the mitochondrion, and directly affecting mitochondrial function and ATP synthesis [52]. Mitochondrial cristae are structured such that OXPHOS complexes are near one another, with Complex V (ATP Synthase) placed at the edge of the cristae with the other complexes located along both sides [54]. This strict shaping of the cristae appears to be fundamental for the creation of a proton gradient, with protons flowing from the complexes to the ATP Synthase [54]. Complex I is an L shaped molecule, with a peripheral arm that protrudes into the mitochondrial matrix, and a membrane arm that sits in the inner membrane [52]. The two functional blocks within the peripheral arm have been termed the N (NADH binding) and Q (ubiquinone binding) modules. The membrane arm consists of the P (proton pumping) module, which in turn has proximal and distal ends that form separately during Complex I assembly. Three of the identified increased proteins are involved in the transmembrane module; NDUFA3 in the proximal portion of the P module; and NDUFB3, and NDUFB11 in the distal portion of the P module [52]. NDUFA12 is located at the interface between modules N and Q, while the decreased NDUFA5 is located in the Q module and is in involved in the earliest subassembly of the module [52]. The specific roles of many of the accessory subunits in Complex I are not yet known, it has been proposed that the eukaryotic supernumerary subunits assist in Complex I biogenesis and stability.

Complex I is the major producer of reactive oxygen species (ROS) within the electron transport chain and thus a significant contributor to cellular oxidative stress. It is to be expected that an increase in ROS may be observed in conjunction with the increase in Complex I protein expression, as well as effects on enzymes involved in redox regulation and homeostasis [55]. Complex I is also particularly sensitive to ROS damage [56], therefore the increased expression of Complex I proteins may be in response to this damage. Further, Complex I is frequently found assembled as a supercomplex with Complex III and IV, and this has been proposed as a method of controlling ROS generation by Complex I [56, 57]. The supercomplex acts to prevent excessive superoxide production during oxidation of NAD-linked substrates because the resulting efficient CoQ channeling helps to maintain the chain in the oxidized state [57]. This supercomplex formation relies on balanced stoichiometric ratios of each complex. Increased expression of Complex I subunits may then affect supercomplex organization, and in turn cause a dramatic enhancement of ROS production by Complex I [57].

Perhaps an indication of defective ATP production by ME/CFS mitochondria, a 2016 ME/CFS study of ATP levels in PBMCs observed that ATP levels were higher and mitochondria cristae more condensed when compared to controls [32]. It was noted the increased ATP was largely from non-mitochondrial sources [32]. Perturbations in mitochondria-shaping proteins and disruption to supercomplex formation and thereby cristae structure have been implicated in various diseases, for example Parkinson’s disease [58]. Indeed, an analysis of the mitochondria-shaping proteins altered in the context of Parkinson’s disease (the vast majority of which were related to the organization of cristae) found many of their binding partners were related to the mitochondria and the proteasome [58]. Also sharing similarity with our ME/CFS findings, the mitochondria-shaping proteins altered in Parkinson’s disease are involved in biological pathways relating to the production and metabolism of ATP, immune response and oxidative stress [58].

## Conclusion

Evidence is mounting that mitochondrial dysfunction plays a significant role in the pathogenesis of ME/CFS. Various studies including emerging proteome studies on a range of different biological samples hold promise in uncovering the underlying pathology in complex illnesses such as ME/CFS. Whether any individual changes in protein expression, or combinations of proteins, could act as a diagnostic biomarker for ME/CFS remains to be evaluated, after further validation experiments are carried out with different and larger ME/CFS cohorts, and with other similarly presenting illnesses. However, a model for deficient ATP production and resulting compensatory mechanisms has been developed [27] and corroborated by this study and provides a plausible explanation for the characteristic post-exertional malaise experienced in ME/CFS.

## Supplementary information

**Additional file 1: Table S1.** STRING functional network interactions of proteins increased and decreased (*italicised*) in relative abundance in eleven ME/CFS subjects when compared to nine matched controls (*P*< 0.01, log_10_ (Fold-Change) > 0.2 and < −0.2)**. Table S2.** STRING functional association networks of proteins increased in abundance in the eleven ME/CFS subjects compared to controls (*P*< 0.01, log_10_ (Fold-Change) > 0.2 and < −0.2) Biological processes, molecular functions, cellular components, KEGG and Reactome pathways, UniProt keywords, PFAM, INTERPRO and SMART protein domains are shown. **Table S3.** STRING functional association networks of proteins decreased in abundance in the eleven ME/CFS subjects compared to controls (*P*< 0.01, log_10_ (Fold-Change) > 0.2 and < −0.2) Biological processes, molecular functions, cellular components, KEGG and Reactome pathways, UniProt keywords, PFAM and INTERPRO protein domains are shown. **Table S4.** Proteins with differential relative abundances after t-test comparison of the ‘ME/CFS’ PCA group with the ‘control’ PCA group (*P* < 0.05, Fold Change > 1.3 and < 0.75)**. Figure S1**. Functional annotation clusters generated from proteins with relatively higher abundance in all ME/CFS versus all controls (n = 38, P < 0.01, log_10_(Fold-Change) > 0.2). **Figure S2.** Functional annotation clusters generated from proteins with relatively higher abundance in all ME/CFS thversus all controls (n = 38, P< 0.01, log_10_(Fold-Change) > 0.2). **Figure S3.** Functional annotation clusters generated from proteins with relatively lower abundance in all ME/CFS versus all controls (n = 22, *P* < 0.01, log_10_(Fold-Change) < −0.2).

## Data Availability

All data generated or analysed during this study are included in this published article [and its supplementary information files]. The full raw datasets analysed during the current study (> 710 gigabyte) are available from the corresponding author on reasonable request.

## Abbreviations

ME/CFS: Myalgic Encephalomyelitis/Chronic Fatigue Syndrome
GO: Gene ontology
PC: Principal component
DDA: Data-dependent acquisition
DIA: Data-independent acquisition
SWATH-MS: Sequential Windows Acquisition of all Theoretical Fragment Ion Spectra-mass spectrometry
LC: Liquid chromatography
CCC: Canadian consensus criteria
ROS: Reactive oxygen species
OXPHOS: Oxidative phosphorylation
ATP: Adenosine triphosphate
PBMC: Peripheral blood mononuclear cells
PPI: Protein-protein interaction
TCA: Tricarboxylic acid cycle
