# Supplementary material for: A SWATH-MS analysis of Myalgic Encephalomyelitis/Chronic Fatigue Syndrome peripheral blood mononuclear cell proteomes reveals mitochondrial dysfunction
Source: J Transl Med. 2020 Sep 24;18:365. doi: 10.1186/s12967-020-02533-3 (PMC7512220; doi:10.1186/s12967-020-02533-3)
Supplement: Supplementary file 1 — Additional file 1: Table S1. STRING functional network interactions of proteins increased and decreased (italicised) in relative abundance in eleven ME/CFS subjects when compared to nine matched controls (P< 0.01, log10 (Fold-Change) > 0.2 and < −0.2). Table S2. STRING functional association networks of proteins increased in abundance in the eleven ME/CFS subjects compared to controls (P< 0.01, log10 (Fold-Change) > 0.2 and < −0.2) Biological processes, molecular functions, cellular components, KEGG and Reactome pathways, UniProt keywords, PFAM, INTERPRO and SMART protein domains are shown. Table S3. STRING functional association networks of proteins decreased in abundance in the eleven ME/CFS subjects compared to controls (P< 0.01, log10 (Fold-Change) > 0.2 and < −0.2) Biological processes, molecular functions, cellular components, KEGG and Reactome pathways, UniProt keywords, PFAM and INTERPRO protein domains are shown. Table S4. Proteins with differential relative abundances after t-test comparison of the ‘ME/CFS’ PCA group with the ‘control’ PCA group (P < 0.05, Fold Change > 1.3 and < 0.75). Figure S1. Functional annotation clusters generated from proteins with relatively higher abundance in all ME/CFS versus all controls (n = 38, P < 0.01, log10(Fold-Change) > 0.2). Figure S2. Functional annotation clusters generated from proteins with relatively higher abundance in all ME/CFS thversus all controls (n = 38, P< 0.01, log10(Fold-Change) > 0.2). Figure S3. Functional annotation clusters generated from proteins with relatively lower abundance in all ME/CFS versus all controls (n = 22, P < 0.01, log10(Fold-Change) < −0.2). [file 12967_2020_2533_MOESM1_ESM.docx]

**Table S1:** STRING functional network interactions of proteins increased and decreased (*italicised*) in relative abundance in eleven ME/CFS subjects when compared to nine matched controls (*P*< 0.01, Log_10_ (Fold-Change) > 0.2 and < -0.2)

| **Protein 1** | **Protein 2** | **Neighborhood on Chromosome** | **Gene Fusion** | **Phylogenetic Cooccurrence** | **Homology** | **Coexpression** | **Experimentally Determined Interaction** | **Database Annotated** | **Automated Textmining** | **Combined Score** |
| --- | --- | --- | --- | --- | --- | --- | --- | --- | --- | --- |
| PSMD14 | PSMB4 | 0 | 0 | 0 | 0 | 0.777 | 0.994 | 0.9 | 0.396 | 0.999 |
| ADRM1 | PSMD14 | 0 | 0 | 0 | 0 | 0.304 | 0.936 | 0.9 | 0.918 | 0.999 |
| ADRM1 | PSMB4 | 0 | 0 | 0 | 0 | 0.275 | 0.957 | 0.9 | 0.401 | 0.997 |
| RPS7 | PWP2 | 0 | 0 | 0 | 0 | 0.123 | 0.734 | 0.9 | 0.097 | 0.976 |
| HIST1H1E | HIST1H1D | 0 | 0 | 0 | 0.978 | 0.651 | 0 | 0.9 | 0.862 | 0.964 |
| PSMD14 | CUL3 | 0 | 0 | 0 | 0 | 0.067 | 0.262 | 0.9 | 0.434 | 0.955 |
| HIST1H1B | HIST1H1E | 0 | 0 | 0 | 0.976 | 0.35 | 0 | 0.9 | 0.894 | 0.933 |
| HIST1H1C | HIST1H1E | 0 | 0 | 0 | 0.979 | 0.348 | 0 | 0.9 | 0.86 | 0.933 |
| HIST1H1B | HIST1H1D | 0 | 0 | 0 | 0.971 | 0.348 | 0 | 0.9 | 0.86 | 0.933 |
| HIST1H1B | HIST1H1A | 0 | 0 | 0 | 0.95 | 0.275 | 0 | 0.9 | 0.931 | 0.927 |
| HIST1H1A | HIST1H1D | 0 | 0 | 0 | 0.952 | 0.235 | 0 | 0.9 | 0.868 | 0.923 |
| HIST1H1C | HIST1H1D | 0 | 0 | 0 | 0.975 | 0.249 | 0 | 0.9 | 0.891 | 0.923 |
| PSMB4 | CUL3 | 0 | 0 | 0 | 0 | 0.069 | 0.083 | 0.9 | 0.198 | 0.922 |
| HIST1H1E | HIST1H1A | 0 | 0 | 0 | 0.961 | 0.236 | 0 | 0.9 | 0.815 | 0.922 |
| HIST1H1C | HIST1H1A | 0 | 0 | 0 | 0.954 | 0.169 | 0 | 0.9 | 0.902 | 0.916 |
| HIST1H1C | HIST1H1B | 0 | 0 | 0 | 0.97 | 0.139 | 0 | 0.9 | 0.892 | 0.912 |
| GCA | CPPED1 | 0 | 0 | 0 | 0 | 0.062 | 0 | 0.9 | 0 | 0.902 |
| PSMG1 | PSMB4 | 0 | 0 | 0 | 0 | 0.069 | 0.807 | 0 | 0.263 | 0.856 |
| GNL2 | PWP2 | 0 | 0 | 0 | 0 | 0.668 | 0 | 0 | 0.394 | 0.79 |
| ADRM1 | RPS7 | 0 | 0 | 0 | 0 | 0.7 | 0 | 0 | 0 | 0.7 |
| STRAP | RPRD2 | 0 | 0 | 0 | 0 | 0 | 0.656 | 0 | 0 | 0.656 |
| PSMD14 | PSMG1 | 0 | 0 | 0 | 0 | 0.624 | 0 | 0 | 0.094 | 0.644 |
| PSMD14 | RPS7 | 0 | 0 | 0 | 0 | 0.361 | 0.413 | 0 | 0.062 | 0.617 |
| WDR77 | SF3A1 | 0 | 0 | 0 | 0 | 0.213 | 0.511 | 0 | 0 | 0.599 |
| WDR37 | PWP2 | 0 | 0 | 0 | 0 | 0.101 | 0 | 0.54 | 0.049 | 0.572 |
| STRAP | PSMD14 | 0 | 0 | 0 | 0 | 0.566 | 0 | 0 | 0 | 0.566 |
| STRAP | WDR77 | 0 | 0 | 0.253 | 0 | 0.19 | 0 | 0 | 0.31 | 0.546 |
| WDR37 | RPS7 | 0 | 0 | 0 | 0 | 0.052 | 0 | 0.54 | 0 | 0.545 |
| NXF1 | GNL2 | 0 | 0 | 0 | 0 | 0.098 | 0.07 | 0 | 0.47 | 0.517 |
| GNL2 | HIST1H1A | 0 | 0 | 0 | 0 | 0 | 0.516 | 0 | 0 | 0.516 |
| SLC25A3 | SLC25A11 | 0 | 0 | 0 | 0.615 | 0.349 | 0 | 0 | 0.543 | 0.481 |
| RPS7 | WDR77 | 0 | 0 | 0 | 0 | 0.449 | 0 | 0 | 0 | 0.449 |
| *HBD* | *HBG2* | *0* | *0* | *0* | *0.974* | *0.558* | *0* | *0.9* | *0.653* | *0.954* |
| *C6orf25* | *GP6* | *0* | *0* | *0* | *0* | *0* | *0* | *0.9* | *0.568* | *0.954* |
| *GP1BB* | *GP9* | *0* | *0* | *0* | *0.781* | *0.107* | *0.379* | *0.9* | *0.937* | *0.952* |
| *GP6* | *GP9* | *0* | *0* | *0* | *0* | *0.066* | *0* | *0* | *0.731* | *0.738* |
| *DNAJB11* | *CANX* | *0* | *0* | *0* | *0* | *0.2* | *0.16* | *0* | *0.434* | *0.586* |
| *GP1BB* | *GP6* | *0* | *0* | *0* | *0* | *0* | *0* | *0* | *0.478* | *0.478* |

**Table S2:** STRING functional association networks of proteins increased in abundance in the eleven ME/CFS subjects compared to controls (*P*< 0.01, Log_10_ (Fold-Change) > 0.2 and < -0.2) Biological processes, molecular functions, cellular components, KEGG and Reactome pathways, UniProt keywords, PFAM, INTERPRO and SMART protein domains are shown.

| Biological Process (GO) | | | | |
| --- | --- | --- | --- | --- |
| GO Term | **Description** | **Count in protein set** | **False discovery rate** | **Matching proteins**  **in network** |
| GO:0098532 | histone H3-K27 trimethylation | 3 of 6 | 0.00059 | HIST1H1C,HIST1H1D,HIST1H1E |
| GO:0016584 | nucleosome positioning | 3 of 9 | 0.00077 | HIST1H1C,HIST1H1D,HIST1H1E |
| GO:0034622 | cellular protein-containing complex assembly | 10 of 832 | 0.00085 | ADRM1,HIST1H1A,HIST1H1B,  HIST1H1C,HIST1H1D,HIST1H1E,  PSMG1,PWP2,SF3A1,STRAP |
| GO:0080182 | histone H3-K4 trimethylation | 3 of 15 | 0.0011 | HIST1H1C,HIST1H1D,HIST1H1E |
| GO:0006334 | nucleosome assembly | 5 of 137 | 0.0014 | HIST1H1A,HIST1H1B,HIST1H1C,  HIST1H1D,HIST1H1E |
| GO:0065003 | protein-containing complex assembly | 11 of 1514 | 0.0077 | ADRM1,CUL3,HIST1H1A,HIST1H1B,  HIST1H1C,HIST1H1D,HIST1H1E,  PSMG1,PWP2,SF3A1,STRAP |
| GO:0006479 | protein methylation | 4 of 127 | 0.0083 | HIST1H1C,HIST1H1D,HIST1H1E,  WDR77 |
| GO:0022613 | ribonucleoprotein complex biogenesis | 6 of 409 | 0.0083 | GNL2,PWP2,RPS7,SF3A1,STRAP,  WDR37 |
| GO:0044085 | cellular component biogenesis | 14 of 2556 | 0.0099 | ADRM1,CUL3,GNL2,HIST1H1A,  HIST1H1B,HIST1H1C,HIST1H1D,  HIST1H1E,PSMG1,PWP2,RPS7,SF3A1,  STRAP,WDR37 |
| GO:0043248 | proteasome assembly | 2 of 15 | 0.0224 | ADRM1,PSMG1 |
| GO:0044248 | cellular catabolic process | 10 of 1646 | 0.0382 | ACAT2,ADRM1,ASRGL1,BDH1,CUL3,  FUNDC2,GAPDHS,PSMB4,PSMD14,  RPS7 |
| Molecular Function (GO) | | | | |
| GO Term | **Description** | **Count in protein set** | **False discovery rate** | **Matching proteins in network** |
| GO:0031490 | chromatin DNA binding | 5 of 87 | 0.0002 | HIST1H1A,HIST1H1B,HIST1H1C,  HIST1H1D,HIST1H1E |
| GO:0070628 | proteasome binding | 3 of 13 | 0.0004 | ADRM1,PSMD14,PSMG1 |
| GO:0061133 | endopeptidase activator activity | 2 of 6 | 0.0074 | ADRM1,PSMD14 |
| Cellular Component (GO) | | | | |
| GO Term | **Description** | **Count in protein set** | **False discovery rate** | **Matching proteins in network** |
| GO:0070013 | intracellular organelle lumen | 27 of 5162 | 2.90E-06 | ACAT2,ADRM1,BDH1,CPPED1,CUL3,  GCA,GNL2,HIST1H1A,HIST1H1B,  HIST1H1C,HIST1H1D,HIST1H1E,  NXF1,PSMB4,PSMD14,PSMG1,PWP2,  RPRD2,RPS7,SAE1,SF3A1,STRAP,TIA1,  TXNRD2,VTI1B,WDR37,WDR77 |
| GO:0031981 | nuclear lumen | 23 of 4030 | 6.05E-06 | ACAT2,ADRM1,BDH1,CUL3,GNL2,  HIST1H1A,HIST1H1B,HIST1H1C,  HIST1H1D,HIST1H1E,NXF1,PSMB4,  PSMD14,PSMG1,PWP2,RPRD2,RPS7,  SAE1,SF3A1,STRAP,TIA1,WDR37,  WDR77 |
| GO:0044446 | intracellular organelle part | 32 of 8882 | 3.77E-05 | ACAT2,ADRM1,ARPC1A,BDH1,  CPPED1,CUL3,FUNDC2,GCA,GNL2,  HIST1H1A,HIST1H1B,HIST1H1C,  HIST1H1D,HIST1H1E,NXF1,PSMB4,  PSMD14,PSMG1,PWP2,RPRD2,RPS7,  SAE1,SF3A1,SLC25A11,SLC25A3,STRAP,  TIA1,TXNRD2,USMG5,VTI1B,WDR37,  WDR77 |
| GO:0000786 | nucleosome | 5 of 106 | 7.01E-05 | HIST1H1A,HIST1H1B,HIST1H1C,  HIST1H1D,HIST1H1E |
| GO:0032991 | protein-containing complex | 23 of 4792 | 7.04E-05 | ADRM1,ARPC1A,CLCC1,CUL3,  HIST1H1A,HIST1H1B,HIST1H1C,  HIST1H1D,HIST1H1E,NXF1,PSMB4,  PSMD14,PWP2,RPRD2,RPS7,SAE1,  SF3A1,STRAP,TIA1,USMG5,VTI1B,  WDR37,WDR77 |
| GO:0044424 | intracellular part | 38 of 13996 | 7.33E-05 | ACAT2,ADRM1,ARF4,ARPC1A,ASRGL1,  BDH1,CLCC1,CPPED1,CUL3,FUNDC2,  GAPDHS,GCA,GNL2,HIST1H1A,  HIST1H1B,HIST1H1C,HIST1H1D,  HIST1H1E,ISYNA1,NXF1,PSMB4,  PSMD14,PSMG1,PWP2,RPRD2,RPS7,  SAE1,SF3A1,SH2D1A,SLC25A11,  SLC25A3,STRAP,TIA1,TXNRD2,USMG5,  VTI1B,WDR37,WDR77 |
| GO:0043231 | intracellular membrane-bounded organelle | 33 of 10365 | 0.00021 | ACAT2,ADRM1,ARF4,BDH1,CLCC1,  CPPED1,CUL3,FUNDC2,GCA,GNL2,  HIST1H1A,HIST1H1B,HIST1H1C,  HIST1H1D,HIST1H1E,NXF1,PSMB4,  PSMD14,PSMG1,PWP2,RPRD2,RPS7,  SAE1,SF3A1,SLC25A11,SLC25A3,STRAP,  TIA1,TXNRD2,USMG5,VTI1B,WDR37,  WDR77 |
| GO:0005719 | nuclear euchromatin | 3 of 24 | 0.00033 | HIST1H1A,HIST1H1C,HIST1H1D |
| GO:0030686 | 90S preribosome | 3 of 30 | 0.00055 | PWP2,RPS7,WDR37 |
| GO:0005829 | cytosol | 21 of 4958 | 0.0011 | ACAT2,ADRM1,ARF4,ARPC1A,  ASRGL1,CPPED1,CUL3,GAPDHS,GCA,  ISYNA1,NXF1,PSMB4,PSMD14,PSMG1,  RPS7,SH2D1A,STRAP,TIA1,TXNRD2,  VTI1B,WDR77 |
| GO:0008541 | proteasome regulatory particle, lid subcomplex | 2 of 8 | 0.002 | ADRM1,PSMD14 |
| GO:0043229 | intracellular organelle | 34 of 12193 | 0.0022 | ACAT2,ADRM1,ARF4,ARPC1A,BDH1,  CLCC1,CPPED1,CUL3,FUNDC2,GCA,  GNL2,HIST1H1A,HIST1H1B,HIST1H1C,  HIST1H1D,HIST1H1E,NXF1,PSMB4,  PSMD14,PSMG1,PWP2,RPRD2,RPS7,SAE1,  SF3A1,SLC25A11,SLC25A3,STRAP,TIA1,  TXNRD2,USMG5,VTI1B,WDR37,WDR77 |
| GO:0000502 | proteasome complex | 3 of 64 | 0.0033 | ADRM1,PSMB4,PSMD14 |
| GO:0005634 | nucleus | 24 of 6892 | 0.0041 | ACAT2,ADRM1,BDH1,CLCC1,CUL3,  GNL2,HIST1H1A,HIST1H1B,HIST1H1C,  HIST1H1D,HIST1H1E,NXF1,PSMB4,  PSMD14,PSMG1,PWP2,RPRD2,RPS7,SAE1,  SF3A1,STRAP,TIA1,WDR37,WDR77 |
| GO:0000790 | nuclear chromatin | 5 of 333 | 0.0042 | HIST1H1A,HIST1H1B,HIST1H1C,  HIST1H1D,HIST1H1E |
| GO:0005654 | nucleoplasm | 15 of 3446 | 0.0095 | ADRM1,BDH1,CUL3,NXF1,PSMB4,  PSMD14,PSMG1,PWP2,RPRD2,RPS7,SAE1,  SF3A1,STRAP,TIA1,WDR77 |
| GO:0032040 | small-subunit processome | 2 of 34 | 0.0159 | PWP2,RPS7 |
| GO:0005720 | nuclear heterochromatin | 2 of 35 | 0.0162 | HIST1H1B,HIST1H1E |
| GO:1902494 | catalytic complex | 8 of 1295 | 0.0195 | ADRM1,CUL3,PSMB4,PSMD14,RPRD2,  SAE1,SF3A1,WDR77 |
| GO:0044444 | cytoplasmic part | 27 of 9377 | 0.0204 | ACAT2,ADRM1,ARF4,ARPC1A,ASRGL1,  BDH1,CLCC1,CPPED1,CUL3,FUNDC2,  GAPDHS,GCA,ISYNA1,NXF1,PSMB4,  PSMD14,PSMG1,RPS7,SH2D1A,SLC25A11,  SLC25A3,STRAP,TIA1,TXNRD2,USMG5,  VTI1B,WDR77 |
| GO:0034774 | secretory granule lumen | 4 of 323 | 0.0214 | CPPED1,GCA,PSMD14,VTI1B |
| GO:0044455 | mitochondrial membrane part | 3 of 203 | 0.0408 | BDH1,FUNDC2,USMG5 |
| KEGG Pathways | | | | |
| Pathway | **Description** | **Count in protein set** | **False discovery rate** | **Matching proteins in network** |
| hsa03050 | Proteasome | 3 of 43 | 0.0039 | ADRM1,PSMB4,PSMD14 |
| hsa00072 | Synthesis and degradation of ketone bodies | 2 of 10 | 0.0048 | ACAT2,BDH1 |
| hsa03008 | Ribosome biogenesis in eukaryotes | 3 of 76 | 0.0064 | GNL2,NXF1,PWP2 |
| hsa00650 | Butanoate metabolism | 2 of 28 | 0.0154 | ACAT2,BDH1 |
| Reactome Pathways | | | | |
| Pathway | **Description** | **Count in protein set** | **False discovery rate** | **Matching proteins in network** |
| HSA-140342 | Apoptosis induced DNA fragmentation | 5 of 13 | 4.45E-08 | HIST1H1A,HIST1H1B,HIST1H1C,  HIST1H1D,HIST1H1E |
| HSA-211227 | Activation of DNA fragmentation factor | 5 of 13 | 4.45E-08 | HIST1H1A,HIST1H1B,HIST1H1C,  HIST1H1D,HIST1H1E |
| HSA-2559584 | Formation of Senescence-Associated Heterochromatin Foci (SAHF) | 5 of 16 | 4.45E-08 | HIST1H1A,HIST1H1B,HIST1H1C,  HIST1H1D,HIST1H1E |
| HSA-109581 | Apoptosis | 7 of 162 | 2.00E-06 | HIST1H1A,HIST1H1B,HIST1H1C,  HIST1H1D,HIST1H1E,PSMB4,PSMD14 |
| HSA-5357801 | Programmed Cell Death | 7 of 165 | 2.00E-06 | HIST1H1A,HIST1H1B,HIST1H1C,  HIST1H1D,HIST1H1E,PSMB4,PSMD14 |
| HSA-75153 | Apoptotic execution phase | 5 of 51 | 3.13E-06 | HIST1H1A,HIST1H1B,HIST1H1C,  HIST1H1D,HIST1H1E |
| HSA-2559586 | DNA Damage/Telomere Stress Induced Senescence | 5 of 61 | 6.20E-06 | HIST1H1A,HIST1H1B,HIST1H1C,  HIST1H1D,HIST1H1E |
| HSA-2262752 | Cellular responses to stress | 8 of 384 | 2.19E-05 | HIST1H1A,HIST1H1B,HIST1H1C,  HIST1H1D,HIST1H1E,PSMB4,PSMD14,  TXNRD2 |
| HSA-8953897 | Cellular responses to external stimuli | 8 of 459 | 7.19E-05 | HIST1H1A,HIST1H1B,HIST1H1C,  HIST1H1D,HIST1H1E,PSMB4,PSMD14,  TXNRD2 |
| HSA-2559583 | Cellular Senescence | 5 of 161 | 0.00042 | HIST1H1A,HIST1H1B,HIST1H1C,  HIST1H1D,HIST1H1E |
| HSA-4641258 | Degradation of DVL | 3 of 54 | 0.0042 | CUL3,PSMB4,PSMD14 |
| HSA-8953854 | Metabolism of RNA | 7 of 652 | 0.0051 | NXF1,PSMB4,PSMD14,PWP2,RPS7,  SF3A1,WDR77 |
| HSA-5658442 | Regulation of RAS by GAPs | 3 of 65 | 0.006 | CUL3,PSMB4,PSMD14 |
| HSA-5632684 | Hedgehog 'on' state | 3 of 82 | 0.0108 | CUL3,PSMB4,PSMD14 |
| HSA-5689603 | UCH proteinases | 3 of 93 | 0.0144 | ADRM1,PSMB4,PSMD14 |
| HSA-70263 | Gluconeogenesis | 2 of 33 | 0.0332 | GAPDHS,SLC25A11 |
| HSA-5358351 | Signaling by Hedgehog | 3 of 143 | 0.0421 | CUL3,PSMB4,PSMD14 |
| UniProt Keywords | | | | |
| Keyword | **Description** | **Count in protein set** | **False discovery rate** | **Matching proteins in network** |
| KW-0007 | Acetylation | 24 of 3335 | 3.03E-08 | ACAT2,ADRM1,ASRGL1,BDH1,  CUL3,GNL2,HIST1H1A,HIST1H1B,  HIST1H1C,HIST1H1D,HIST1H1E,  NXF1,PSMB4,PSMG1,RPRD2,RPS7,  SAE1,SF3A1,SH2D1A,SLC25A11,  SLC25A3,TIA1,USMG5,VTI1B |
| KW-0164 | Citrullination | 5 of 65 | 1.14E-05 | HIST1H1A,HIST1H1B,HIST1H1C,  HIST1H1D,HIST1H1E |
| KW-0379 | Hydroxylation | 5 of 136 | 0.00025 | HIST1H1A,HIST1H1B,HIST1H1C,  HIST1H1D,HIST1H1E |
| KW-0647 | Proteasome | 3 of 50 | 0.0036 | ADRM1,PSMB4,PSMD14 |
| KW-0853 | WD repeat | 5 of 279 | 0.0041 | ARPC1A,PWP2,STRAP,WDR37,WDR77 |
| KW-0597 | Phosphoprotein | 26 of 8066 | 0.0108 | ADRM1,ARF4,BDH1,CLCC1,CPPED1,  CUL3,FUNDC2,GNL2,HIST1H1A,  HIST1H1B,HIST1H1C,HIST1H1D,  HIST1H1E,NXF1,PSMB4,PSMD14,  PSMG1,PWP2,RPRD2,SAE1,SF3A1,  SLC25A11,SLC25A3,STRAP,VTI1B,  WDR77 |
| KW-0158 | Chromosome | 5 of 409 | 0.0164 | HIST1H1A,HIST1H1B,HIST1H1C,  HIST1H1D,HIST1H1E |
| KW-0488 | Methylation | 7 of 959 | 0.0282 | HIST1H1B,HIST1H1C,HIST1H1E,  NXF1,RPRD2,SLC25A3,VTI1B |
| PFAM Protein Domains | | | | |
| Domain | **Description** | **Count in protein set** | **False discovery rate** | **Matching proteins in network** |
| PF00538 | linker histone H1 and H5 family | 5 of 11 | 4.03E-09 | HIST1H1A,HIST1H1B,HIST1H1C,  HIST1H1D,HIST1H1E |
| PF00400 | WD domain, G-beta repeat | 5 of 224 | 0.0017 | ARPC1A,PWP2,STRAP,WDR37,  WDR77 |
| INTERPRO Protein Domains and Features | | | | |
| Domain | **Description** | **Count in protein set** | **False discovery rate** | **Matching proteins in network** |
| IPR005819 | Histone H5 | 5 of 8 | 2.46E-09 | HIST1H1A,HIST1H1B,HIST1H1C,  HIST1H1D,HIST1H1E |
| IPR005818 | Linker histone H1/H5, domain H15 | 5 of 13 | 8.14E-09 | HIST1H1A,HIST1H1B,HIST1H1C,  HIST1H1D,HIST1H1E |
| IPR036390 | Winged helix DNA-binding domain superfamily | 6 of 209 | 0.0001 | CUL3,HIST1H1A,HIST1H1B,  HIST1H1C,HIST1H1D,HIST1H1E |
| IPR036388 | Winged helix-like DNA-binding domain superfamily | 6 of 230 | 0.00013 | CUL3,HIST1H1A,HIST1H1B,  HIST1H1C,HIST1H1D,HIST1H1E |
| IPR017986 | WD40-repeat-containing domain | 5 of 243 | 0.0021 | ARPC1A,PWP2,STRAP,WDR37,  WDR77 |
| IPR001680 | WD40 repeat | 5 of 269 | 0.0027 | ARPC1A,PWP2,STRAP,WDR37,  WDR77 |
| IPR019775 | WD40 repeat, conserved site | 4 of 164 | 0.0041 | PWP2,STRAP,WDR37,WDR77 |
| IPR015943 | WD40/YVTN repeat-like-containing domain superfamily | 5 of 333 | 0.0054 | ARPC1A,PWP2,STRAP,WDR37,  WDR77 |
| IPR020472 | G-protein beta WD-40 repeat | 3 of 85 | 0.0068 | PWP2,STRAP,WDR37 |
| IPR029055 | Nucleophile aminohydrolases, N-terminal | 2 of 35 | 0.0214 | ASRGL1,PSMB4 |
| IPR036322 | WD40-repeat-containing domain superfamily | 4 of 285 | 0.0214 | ARPC1A,STRAP,WDR37,WDR77 |
| IPR036291 | NAD(P)-binding domain superfamily | 3 of 155 | 0.0273 | BDH1,GAPDHS,ISYNA1 |
| IPR018108 | Mitochondrial substrate/solute carrier | 2 of 54 | 0.0377 | SLC25A11,SLC25A3 |
| IPR023395 | Mitochondrial carrier domain superfamily | 2 of 54 | 0.0377 | SLC25A11,SLC25A3 |
| SMART Protein Domains | | | | |
| Domain | **Description** | **Count in protein set** | **False discovery rate** | **Matching proteins in network** |
| SM00526 | Domain in histone families 1 and 5 | 5 of 13 | 2.80E-09 | HIST1H1A,HIST1H1B,HIST1H1C,  HIST1H1D,HIST1H1E |
| SM00320 | WD40 repeats | 5 of 274 | 0.0015 | ARPC1A,PWP2,STRAP,WDR37,WDR77 |

**Table S3:** STRING functional association networks of proteins decreased in abundance in the eleven ME/CFS subjects compared to controls (*P*< 0.01, Log_10_ (Fold-Change) > 0.2 and < -0.2) Biological processes, molecular functions, cellular components, KEGG and Reactome pathways, UniProt keywords, PFAM and INTERPRO protein domains are shown.

| Biological Process (GO) | | | | |
| --- | --- | --- | --- | --- |
| GO Term | **Description** | **Count in protein set** | **False discovery rate** | **Matching proteins in network** |
| GO:0007596 | blood coagulation | 6 of 288 | 0.00045 | C6orf25,GP1BB,GP6,GP9,HBD,HBG2 |
| GO:0030168 | platelet activation | 4 of 120 | 0.0016 | C6orf25,GP1BB,GP6,GP9 |
| GO:0015671 | oxygen transport | 2 of 15 | 0.0139 | HBD,HBG2 |
| GO:0007597 | blood coagulation, intrinsic pathway | 2 of 17 | 0.0155 | GP1BB,GP9 |
| GO:0007166 | cell surface receptor signaling pathway | 9 of 2198 | 0.0226 | ARL3,C6orf25,CANX,GP1BB,GP6,PLXNA4,PPIA,RASA1,SLA2 |
| GO:0050896 | response to stimulus | 17 of 7824 | 0.0245 | ARL3,BLMH,C6orf25,CANX,CPNE2,DNAJB11,GP1BB,GP6,GP9,HBD,HBG2,MAPK13,NAMPT,PLXNA4,PPIA,RASA1,SLA2 |
| GO:0001775 | cell activation | 6 of 1024 | 0.0368 | C6orf25,GP1BB,GP6,GP9,PPIA,SLA2 |
| Molecular Function (GO) | | | | |
| GO Term | **Description** | **Count in protein set** | **False discovery rate** | **Matching proteins in network** |
| GO:0005344 | oxygen carrier activity | 2 of 14 | 0.0183 | HBD,HBG2 |
| GO:0051082 | unfolded protein binding | 3 of 106 | 0.0183 | CANX,DNAJB11,PPIA |
| GO:0019825 | oxygen binding | 2 of 36 | 0.0352 | HBD,HBG2 |
| Cellular Component (GO) | | |  |  |
| GO Term | **Description** | **Count in protein set** | **False discovery rate** | **Matching proteins in network** |
| GO:0005833 | hemoglobin complex | 2 of 12 | 0.0154 | HBD,HBG2 |
| KEGG Pathways | | | | |
| Pathway | **Description** | **Count in protein set** | **False discovery rate** | **Matching proteins in network** |
| hsa04611 | Platelet activation | 4 of 123 | 0.00066 | GP1BB,GP6,GP9,MAPK13 |
| hsa04512 | ECM-receptor interaction | 3 of 81 | 0.0033 | GP1BB,GP6,GP9 |
| Reactome Pathways | | | | |
| Pathway | **Description** | **Count in protein set** | **False discovery rate** | **Matching proteins in network** |
| HSA-75892 | Platelet Adhesion to exposed collagen | 3 of 15 | 0.00012 | GP1BB,GP6,GP9 |
| HSA-109582 | Hemostasis | 7 of 601 | 0.00018 | C6orf25,GP1BB,GP6,GP9,HBD,HBG2,PPIA |
| HSA-76002 | Platelet activation, signaling and aggregation | 5 of 256 | 0.00034 | C6orf25,GP1BB,GP6,GP9,PPIA |
| HSA-430116 | GP1b-IX-V activation signalling | 2 of 11 | 0.0027 | GP1BB,GP9 |
| HSA-140837 | Intrinsic Pathway of Fibrin Clot Formation | 2 of 22 | 0.0077 | GP1BB,GP9 |
| HSA-114604 | GPVI-mediated activation cascade | 2 of 34 | 0.0145 | C6orf25,GP6 |
| HSA-140877 | Formation of Fibrin Clot (Clotting Cascade) | 2 of 39 | 0.0145 | GP1BB,GP9 |
| HSA-76009 | Platelet Aggregation (Plug Formation) | 2 of 37 | 0.0145 | GP1BB,GP9 |
| HSA-447115 | Interleukin-12 family signaling | 2 of 57 | 0.0258 | CANX,PPIA |
| UniProt Keywords | | | | |
| Keyword | **Description** | **Count in protein set** | **False discovery rate** | **Matching proteins in network** |
| KW-0087 | Bernard Soulier syndrome | 2 of 3 | 0.001 | GP1BB,GP9 |
| KW-0094 | Blood coagulation | 3 of 46 | 0.001 | GP1BB,GP6,GP9 |
| KW-0561 | Oxygen transport | 2 of 11 | 0.002 | HBD,HBG2 |
| KW-0007 | Acetylation | 10 of 3335 | 0.031 | BLMH,CANX,EIF2S2,HBD,HBG2,NAMPT,PLXNA4,PPIA,RASA1,ZFR |
| KW-0597 | Phosphoprotein | 16 of 8066 | 0.0398 | ARL3,C1orf123,C6orf25,CANX,DNAJB11,EIF2S2,ENSG00000263264,GP1BB,HBD,HBG2,MAPK13,NAMPT,PPIA,RASA1,SLA2,ZFR |
| PFAM Protein Domains | | | | |
| Domain | **Description** | **Count in protein set** | **False discovery rate** | **Matching proteins in network** |
| PF00042 | Globin | 2 of 13 | 0.004 | HBD,HBG2 |
| INTERPRO Protein Domains and Features | | | | |
| Domain | **Description** | **Count in protein set** | **False discovery rate** | **Matching proteins in network** |
| IPR002337 | Haemoglobin, beta-type | 2 of 4 | 0.0013 | HBD,HBG2 |
| IPR000971 | Globin | 2 of 11 | 0.0034 | HBD,HBG2 |
| IPR009050 | Globin-like superfamily | 2 of 11 | 0.0034 | HBD,HBG2 |
| IPR012292 | Globin/Protoglobin | 2 of 12 | 0.0034 | HBD,HBG2 |
| IPR000372 | Leucine-rich repeat N-terminal domain | 2 of 54 | 0.0265 | GP1BB,GP9 |

**Table S4: Proteins with differential relative abundances after t-test comparison of the ‘ME/CFS’ PCA group with the ‘control’ PCA group (*P* < 0.05, Fold Change > 1.3 and < 0.75)**

| **GI Accession** | **Protein name** | ***P*-value** | **Fold-Change** | **Log_10_(Fold-Change)** |
| --- | --- | --- | --- | --- |
| 41281564 | WD repeat-containing protein 37 | 1.63E-05 | 1.84 | 0.26 |
| 25282407 | interferon regulatory factor 9 | 2.95E-05 | 1.52 | 0.18 |
| 7706495 | dnaJ homolog subfamily B member 11 precursor | 3.79E-05 | 0.49 | -0.31 |
| 4506085 | mitogen-activated protein kinase 13 | 5.85E-05 | 0.39 | -0.41 |
| 28178832 | isocitrate dehydrogenase [NADP], mitochondrial precursor | 0.00014 | 0.59 | -0.23 |
| 22538467 | proteasome subunit beta type-4 | 0.00016 | 1.85 | 0.27 |
| 115270970 | chloride channel CLIC-like protein 1 isoform 1 precursor | 0.00016 | 1.93 | 0.28 |
| 7706497 | UMP-CMP kinase isoform a | 0.00021 | 0.55 | -0.26 |
| 24371248 | FUN14 domain-containing protein 2 | 0.00023 | 1.91 | 0.28 |
| 4505361 | NADH dehydrogenase [ubiquinone] 1 beta subcomplex subunit 3 | 0.00034 | 1.60 | 0.20 |
| 10863927 | peptidyl-prolyl cis-trans isomerase A | 0.00037 | 0.45 | -0.35 |
| 56676393 | rho GDP-dissociation inhibitor 2 | 0.00038 | 1.54 | 0.19 |
| 11496885 | PDZ and LIM domain protein 7 isoform 1 | 0.00041 | 0.59 | -0.23 |
| 5031977 | nicotinamide phosphoribosyltransferase precursor | 0.00046 | 0.41 | -0.39 |
| 5031987 | peptidyl-prolyl cis-trans isomerase F, mitochondrial precursor | 0.00046 | 0.38 | -0.43 |
| 4757774 | ADP-ribosylation factor-like protein 3 | 0.00047 | 0.47 | -0.32 |
| 9507215 | tubulin alpha-8 chain isoform 1 | 0.00050 | 0.63 | -0.20 |
| 5454166 | vesicle transport through interaction with t-SNAREs homolog 1B | 0.00054 | 2.17 | 0.34 |
| 14149916 | src-like-adapter 2 isoform a | 0.00056 | 0.54 | -0.27 |
| 23821044 | E3 ubiquitin-protein ligase ZNRF2 | 0.00058 | 1.61 | 0.21 |
| 4506133 | phosphoribosyl pyrophosphate synthase-associated protein 2 isoform 1 | 0.00059 | 1.54 | 0.19 |
| 34101286 | zinc finger RNA-binding protein | 0.00059 | 0.50 | -0.30 |
| 4507729 | tubulin beta-2A chain | 0.00063 | 0.58 | -0.23 |
| 29788768 | tubulin beta-2B chain | 0.00063 | 0.58 | -0.23 |
| 143770741 | platelet glycoprotein VI isoform 1 precursor | 0.00064 | 0.56 | -0.25 |
| 20986512 | mitogen-activated protein kinase 14 isoform 2 | 0.00065 | 0.70 | -0.15 |
| 4507143 | sorting nexin-3 isoform a | 0.00066 | 1.40 | 0.15 |
| 8923541 | UPF0587 protein C1orf123 | 0.00067 | 0.26 | -0.58 |
| 7019419 | nucleolar GTP-binding protein 2 | 0.00069 | 2.63 | 0.42 |
| 148727341 | serine-threonine kinase receptor-associated protein | 0.00070 | 1.66 | 0.22 |
| 4503513 | eukaryotic translation initiation factor 3 subunit I | 0.00077 | 1.46 | 0.16 |
| 6912388 | grancalcin | 0.00077 | 1.92 | 0.28 |
| 6715607 | hemoglobin subunit gamma-2 | 0.00088 | 0.25 | -0.60 |
| 4557367 | bleomycin hydrolase | 0.00090 | 0.40 | -0.40 |
| 44680136 | D-beta-hydroxybutyrate dehydrogenase, mitochondrial precursor | 0.00090 | 2.89 | 0.46 |
| 14149777 | haloacid dehalogenase-like hydrolase domain-containing protein 2 | 0.00092 | 0.44 | -0.36 |
| 384551649 | proto-oncogene vav isoform 3 | 0.00097 | 1.65 | 0.22 |
| 7661844 | coiled-coil domain-containing protein 22 | 0.00099 | 1.56 | 0.19 |
| 4759034 | eukaryotic peptide chain release factor subunit 1 | 0.0011 | 0.53 | -0.28 |
| 5031973 | protein disulfide-isomerase A6 precursor | 0.0012 | 0.73 | -0.14 |
| 264681563 | xaa-Pro aminopeptidase 1 isoform 1 | 0.0012 | 1.43 | 0.16 |
| 30181236 | copine-2 | 0.0012 | 0.34 | -0.47 |
| 4506195 | proteasome subunit beta type-2 isoform 1 | 0.0013 | 1.55 | 0.19 |
| 66933005 | calnexin precursor | 0.0013 | 0.56 | -0.25 |
| 5453954 | serine/threonine-protein phosphatase 2A 56 kDa regulatory subunit delta isoform isoform 1 | 0.0016 | 1.50 | 0.18 |
| 28373194 | proteasomal ubiquitin receptor ADRM1 precursor | 0.0017 | 3.07 | 0.49 |
| 12545406 | ras GTPase-activating protein 1 isoform 2 | 0.0017 | 0.56 | -0.25 |
| 19913385 | protein G6b isoform G6b-G precursor | 0.0017 | 0.30 | -0.52 |
| 4826964 | UV excision repair protein RAD23 homolog A isoform 1 | 0.0018 | 1.58 | 0.20 |
| 13129110 | methylosome protein 50 | 0.0021 | 1.86 | 0.27 |
| 7657532 | protein S100-A6 | 0.0021 | 1.38 | 0.14 |
| 63055049 | phosphoglucomutase-2 | 0.0022 | 1.37 | 0.14 |
| 4504073 | platelet glycoprotein Ib beta chain precursor | 0.0023 | 0.50 | -0.30 |
| 145309311 | probable ubiquitin carboxyl-terminal hydrolase FAF-X isoform 4 | 0.0024 | 0.66 | -0.18 |
| 4505023 | proteasome assembly chaperone 1 isoform a | 0.0024 | 3.57 | 0.55 |
| 7705558 | inositol-3-phosphate synthase 1 isoform 1 | 0.0025 | 1.82 | 0.26 |
| 5031981 | 26S proteasome non-ATPase regulatory subunit 14 | 0.0025 | 2.24 | 0.35 |
| 29826335 | eukaryotic translation initiation factor 2 subunit 2 | 0.0026 | 0.45 | -0.34 |
| 145275202 | isoaspartyl peptidase/L-asparaginase | 0.0027 | 1.50 | 0.18 |
| 4503165 | cullin-3 isoform 1 | 0.0027 | 2.91 | 0.46 |
| 4759300 | vesicle-associated membrane protein 3 | 0.0027 | 1.64 | 0.21 |
| 29789060 | C2 domain-containing protein 5 | 0.0027 | 0.61 | -0.21 |
| 54792069 | small ubiquitin-related modifier 2 isoform a precursor | 0.0027 | 1.41 | 0.15 |
| 4507793 | ubiquitin-conjugating enzyme E2 N | 0.0030 | 1.55 | 0.19 |
| 4504077 | platelet glycoprotein IX precursor | 0.0032 | 0.41 | -0.39 |
| 311893365 | calpain-1 catalytic subunit | 0.0032 | 0.63 | -0.20 |
| 499589768 | neutral alpha-glucosidase AB isoform 6 | 0.0033 | 1.36 | 0.14 |
| 7706663 | COMM domain-containing protein 10 | 0.0034 | 1.49 | 0.17 |
| 148539872 | acetyl-CoA acetyltransferase, cytosolic | 0.0034 | 1.64 | 0.21 |
| 57013276 | tubulin alpha-1B chain | 0.0034 | 0.73 | -0.14 |
| 313569797 | arylsulfatase A isoform a precursor | 0.0036 | 1.47 | 0.17 |
| 514052670 | actin-related protein 2/3 complex subunit 3 | 0.0037 | 0.65 | -0.19 |
| 48762926 | periodic tryptophan protein 2 homolog | 0.0038 | 2.49 | 0.40 |
| 62912479 | HLA class I histocompatibility antigen, alpha chain E precursor | 0.0038 | 0.57 | -0.24 |
| 14389309 | tubulin alpha-1C chain | 0.0038 | 0.73 | -0.13 |
| 5453722 | acyl-protein thioesterase 1 | 0.0038 | 1.67 | 0.22 |
| 157738645 | plexin-A4 isoform 1 precursor | 0.0039 | 0.34 | -0.47 |
| 183396804 | regulation of nuclear pre-mRNA domain-containing protein 2 | 0.0040 | 2.52 | 0.40 |
| 4506923 | SH2 domain-containing protein 1A isoform 1 | 0.0040 | 2.01 | 0.30 |
| 20149322 | acylphosphatase-2 | 0.0040 | 1.56 | 0.19 |
| 190014603 | TBC1 domain family member 13 | 0.0041 | 0.48 | -0.31 |
| 259155315 | mitochondrial 2-oxoglutarate/malate carrier protein isoform 2 | 0.0042 | 1.78 | 0.25 |
| 5453597 | F-actin-capping protein subunit alpha-1 | 0.0045 | 0.61 | -0.21 |
| 25777612 | 26S proteasome non-ATPase regulatory subunit 3 | 0.0045 | 1.64 | 0.22 |
| 109134349 | coatomer subunit gamma-2 | 0.0045 | 1.35 | 0.13 |
| 17986001 | major histocompatibility complex, class I, B precursor | 0.0048 | 0.61 | -0.21 |
| 410173533 | PREDICTED: uncharacterized protein LOC100996504 | 0.0048 | 0.47 | -0.32 |
| 4885165 | cystatin-A | 0.0048 | 1.49 | 0.17 |
| 82546879 | GTP-binding protein 1 | 0.0048 | 0.48 | -0.32 |
| 325651836 | SWI/SNF-related matrix-associated actin-dependent regulator of chromatin subfamily A member 5 | 0.0050 | 1.58 | 0.20 |
| 169160905 | PREDICTED: uncharacterized protein LOC128322 | 0.0050 | 2.70 | 0.43 |
| 66392203 | NME1-NME2 protein | 0.0050 | 0.64 | -0.19 |
| 188219591 | nucleolysin TIA-1 isoform p40 isoform 2 | 0.0050 | 1.94 | 0.29 |
| 367460087 | myosin-10 isoform 2 | 0.0051 | 0.75 | -0.13 |
| 373432684 | ubiquitin-conjugating enzyme E2 L3 isoform 4 | 0.0052 | 0.71 | -0.15 |
| 88900491 | neutral alpha-glucosidase AB isoform 3 precursor | 0.0052 | 1.36 | 0.13 |
| 15487670 | nuclear RNA export factor 1 isoform 1 | 0.0052 | 1.70 | 0.23 |
| 4505591 | peroxiredoxin-1 | 0.0053 | 1.47 | 0.17 |
| 225543288 | SUMO-activating enzyme subunit 1 isoform c | 0.0053 | 1.88 | 0.27 |
| 8670546 | cytohesin-2 isoform 1 | 0.0054 | 0.55 | -0.26 |
| 17921989 | tubulin alpha-4A chain isoform 1 | 0.0055 | 0.72 | -0.14 |
| 24797067 | HLA class I histocompatibility antigen, A-1 alpha chain precursor | 0.0056 | 0.63 | -0.20 |
| 4557797 | nucleoside diphosphate kinase A isoform b | 0.0057 | 0.65 | -0.19 |
| 5730041 | suppressor of G2 allele of SKP1 homolog isoform SGT1A | 0.0058 | 1.43 | 0.16 |
| 39725636 | transmembrane emp24 domain-containing protein 9 precursor | 0.0059 | 1.41 | 0.15 |
| 116008188 | myosin light chain kinase, smooth muscle isoform 2 | 0.0059 | 0.75 | -0.13 |
| 4502205 | ADP-ribosylation factor 4 | 0.0059 | 1.92 | 0.28 |
| 47132595 | phosphate carrier protein, mitochondrial isoform b precursor | 0.0060 | 2.22 | 0.35 |
| 91208418 | transcription elongation regulator 1 isoform 2 | 0.0061 | 0.56 | -0.25 |
| 4885373 | histone H1.1 | 0.0062 | 2.11 | 0.32 |
| 4758700 | MAP kinase-activated protein kinase 3 | 0.0062 | 1.82 | 0.26 |
| 18765694 | dipeptidyl peptidase 4 | 0.0064 | 1.40 | 0.15 |
| 19923430 | COMM domain-containing protein 5 | 0.0065 | 2.55 | 0.41 |
| 125988409 | protein Red | 0.0065 | 0.51 | -0.29 |
| 14249348 | thioredoxin domain-containing protein 17 | 0.0065 | 1.58 | 0.20 |
| 4506643 | 60S ribosomal protein L37a | 0.0066 | 1.82 | 0.26 |
| 4504351 | hemoglobin subunit delta | 0.0067 | 0.44 | -0.36 |
| 4885375 | histone H1.2 | 0.0068 | 1.90 | 0.28 |
| 4557469 | AP-2 complex subunit beta isoform b | 0.0070 | 1.31 | 0.12 |
| 4503363 | dolichol-phosphate mannosyltransferase | 0.0071 | 0.46 | -0.33 |
| 108936958 | DDB1- and CUL4-associated factor 7 | 0.0072 | 0.55 | -0.26 |
| 17149834 | sortilin isoform 1 preproprotein | 0.0073 | 0.50 | -0.30 |
| 93277074 | ribonuclease P protein subunit p25 | 0.0073 | 0.47 | -0.33 |
| 21264315 | EH domain-containing protein 4 | 0.0074 | 0.62 | -0.21 |
| 450558 | platelet-activating factor acetylhydrolase IB subunit beta isoform a | 0.0075 | 0.20 | -0.71 |
| 4506741 | 40S ribosomal protein S7 | 0.0076 | 1.76 | 0.24 |
| 18450371 | receptor-type tyrosine-protein phosphatase alpha isoform 2 precursor | 0.0076 | 0.64 | -0.19 |
| 23238222 | COP9 signalosome complex subunit 3 isoform 1 | 0.0077 | 1.64 | 0.21 |
| 5901896 | ATP synthase subunit epsilon, mitochondrial | 0.0079 | 1.70 | 0.23 |
| 4505773 | prohibitin | 0.0079 | 1.40 | 0.15 |
| 4885379 | histone H1.4 | 0.0080 | 1.85 | 0.27 |
| 365733590 | CD48 antigen isoform 2 precursor | 0.0080 | 1.64 | 0.22 |
| 20357529 | guanine nucleotide-binding protein G(I)/G(S)/G(T) subunit beta-2 | 0.0080 | 1.45 | 0.16 |
| 38373671 | protein transport protein Sec24C | 0.0081 | 0.68 | -0.17 |
| 5032087 | splicing factor 3A subunit 1 isoform 1 | 0.0081 | 1.65 | 0.22 |
| 4758638 | peroxiredoxin-6 | 0.0081 | 0.66 | -0.18 |
| 41281905 | fermitin family homolog 3 long form | 0.0081 | 0.68 | -0.17 |
| 7657116 | glyceraldehyde-3-phosphate dehydrogenase, testis-specific | 0.0081 | 2.31 | 0.36 |
| 194306629 | endoplasmic reticulum aminopeptidase 2 | 0.0082 | 0.60 | -0.22 |
| 20544151 | chromobox protein homolog 3 | 0.0083 | 1.55 | 0.19 |
| 7706708 | testis-expressed sequence 264 protein isoform 1 precursor | 0.0083 | 1.52 | 0.18 |
| 13699256 | nuclear inhibitor of protein phosphatase 1 isoform alpha | 0.0084 | 1.49 | 0.17 |
| 4885377 | histone H1.3 | 0.0086 | 1.95 | 0.29 |
| 206725526 | signal-induced proliferation-associated protein 1 | 0.0087 | 1.57 | 0.20 |
| 7705827 | GTP-binding protein SAR1b | 0.0087 | 0.63 | -0.20 |
| 5031635 | cofilin-1 | 0.0088 | 1.45 | 0.16 |
| 67191208 | polyubiquitin-C | 0.0089 | 1.47 | 0.17 |
| 189181666 | beta-hexosaminidase subunit alpha preproprotein | 0.0090 | 0.55 | -0.26 |
| 50726979 | low affinity immunoglobulin gamma Fc region receptor III-A isoform a precursor | 0.0091 | 0.43 | -0.37 |
| 395394071 | thiosulfate sulfurtransferase | 0.0092 | 0.65 | -0.19 |
| 38524622 | formin-binding protein 1 | 0.0093 | 1.40 | 0.15 |
| 56243522 | syntenin-1 isoform 1 | 0.0094 | 1.79 | 0.25 |
| 15431310 | keratin, type I cytoskeletal 14 | 0.0095 | 1.45 | 0.16 |
| 23308697 | signal recognition particle receptor subunit alpha isoform 1 | 0.0097 | 1.56 | 0.19 |
| 4758862 | eukaryotic translation elongation factor 1 epsilon-1 isoform 1 | 0.0098 | 1.80 | 0.25 |
| 190684694 | ubiquitin carboxyl-terminal hydrolase 8 | 0.0098 | 0.50 | -0.30 |
| 94721250 | vesicle-associated membrane protein-associated protein A isoform 1 | 0.0099 | 0.55 | -0.26 |
| 38044290 | zinc finger CCHC domain-containing protein 8 | 0.0099 | 0.63 | -0.20 |
| 195972866 | keratin, type I cytoskeletal 10 | 0.010 | 1.53 | 0.19 |
| 71361682 | nuclear mitotic apparatus protein 1 | 0.010 | 1.32 | 0.12 |
| 4502057 | arachidonate 5-lipoxygenase isoform 1 | 0.010 | 3.06 | 0.49 |
| 4758302 | enhancer of rudimentary homolog | 0.010 | 1.51 | 0.18 |
| 4758964 | cytohesin-1 isoform 1 | 0.010 | 0.45 | -0.34 |
| 24430192 | keratin, type I cytoskeletal 16 | 0.010 | 1.44 | 0.16 |
| 88758615 | integrin alpha-IIb preproprotein | 0.010 | 0.62 | -0.21 |
| 48255966 | UTP--glucose-1-phosphate uridylyltransferase isoform a | 0.010 | 1.38 | 0.14 |
| 32171247 | cyclin-Y isoform 1 | 0.011 | 0.39 | -0.41 |
| 119372308 | beta-galactosidase isoform a preproprotein | 0.011 | 0.73 | -0.14 |
| 167614488 | TBC1 domain family member 10B | 0.011 | 1.54 | 0.19 |
| 4885381 | histone H1.5 | 0.011 | 2.08 | 0.32 |
| 87240000 | methylenetetrahydrofolate reductase | 0.011 | 1.76 | 0.25 |
| 7657058 | translation initiation factor eIF-2B subunit beta | 0.011 | 2.31 | 0.36 |
| 18034692 | choline transporter-like protein 1 | 0.011 | 0.36 | -0.45 |
| 9910542 | GTP-binding protein SAR1a | 0.011 | 0.68 | -0.17 |
| 153082722 | intercellular adhesion molecule 2 precursor | 0.011 | 0.43 | -0.37 |
| 7657056 | EH domain-containing protein 3 | 0.011 | 0.73 | -0.14 |
| 4758032 | coatomer subunit beta' | 0.011 | 1.35 | 0.13 |
| 19913412 | major vault protein | 0.011 | 1.54 | 0.19 |
| 4557493 | cleavage stimulation factor subunit 2 | 0.011 | 1.65 | 0.22 |
| 5803165 | protein transport protein Sec61 subunit beta | 0.011 | 1.63 | 0.21 |
| 10835240 | high mobility group nucleosome-binding domain-containing protein 4 | 0.011 | 1.81 | 0.26 |
| 62420888 | dipeptidyl peptidase 2 preproprotein | 0.011 | 1.59 | 0.20 |
| 153251272 | calcineurin-like phosphoesterase domain-containing protein 1 isoform b | 0.011 | 1.63 | 0.21 |
| 61743954 | neuroblast differentiation-associated protein AHNAK isoform 1 | 0.011 | 1.60 | 0.21 |
| 374253794 | B-cell receptor-associated protein 31 isoform b | 0.012 | 1.40 | 0.14 |
| 193083110 | poly(rC)-binding protein 2 isoform e | 0.012 | 1.46 | 0.17 |
| 300360515 | actin-related protein 2/3 complex subunit 1A isoform 2 | 0.012 | 1.71 | 0.23 |
| 18375632 | large proline-rich protein BAG6 isoform b | 0.012 | 0.75 | -0.13 |
| 4507785 | SUMO-conjugating enzyme UBC9 | 0.012 | 0.54 | -0.26 |
| 23110944 | proteasome subunit alpha type-6 | 0.012 | 1.50 | 0.18 |
| 166795301 | prenylcysteine oxidase 1 precursor | 0.012 | 0.55 | -0.26 |
| 21070997 | stromal interaction molecule 1 isoform 2 precursor | 0.012 | 0.74 | -0.13 |
| 4557431 | T-cell surface glycoprotein CD3 zeta chain isoform 2 precursor | 0.012 | 0.50 | -0.31 |
| 86793036 | complement receptor type 1 isoform F precursor | 0.012 | 0.59 | -0.23 |
| 223468571 | 39S ribosomal protein L23, mitochondrial | 0.012 | 0.45 | -0.35 |
| 4758152 | mitochondrial import inner membrane translocase subunit Tim8 A isoform 1 | 0.012 | 0.47 | -0.33 |
| 5032193 | TNF receptor-associated factor 1 isoform a | 0.013 | 0.39 | -0.41 |
| 11128019 | cytochrome c | 0.013 | 0.56 | -0.25 |
| 186972143 | tripeptidyl-peptidase 2 | 0.013 | 1.51 | 0.18 |
| 344179098 | ATP synthase mitochondrial F1 complex assembly factor 1 isoform 2 precursor | 0.013 | 0.60 | -0.22 |
| 134133265 | FYVE, RhoGEF and PH domain-containing protein 3 | 0.013 | 1.83 | 0.26 |
| 153791497 | rootletin | 0.013 | 0.60 | -0.22 |
| 4507171 | SPARC precursor | 0.013 | 0.26 | -0.59 |
| 25014109 | selenoprotein H | 0.013 | 1.62 | 0.21 |
| 4557263 | ADP-ribosylarginine hydrolase | 0.013 | 0.67 | -0.18 |
| 298919181 | nuclear receptor corepressor 1 isoform 3 | 0.013 | 0.48 | -0.32 |
| 7705773 | endophilin-B1 isoform 1 | 0.014 | 1.46 | 0.17 |
| 22035672 | thioredoxin reductase 2, mitochondrial precursor | 0.014 | 2.18 | 0.34 |
| 330340389 | up-regulated during skeletal muscle growth protein 5 | 0.014 | 1.79 | 0.25 |
| 194328699 | ribosomal RNA small subunit methyltransferase NEP1 | 0.014 | 1.78 | 0.25 |
| 27436946 | lamin isoform A | 0.014 | 1.38 | 0.14 |
| 7661936 | scaffold attachment factor B2 | 0.014 | 1.50 | 0.18 |
| 41393551 | glutamine-dependent NAD(+) synthetase | 0.014 | 1.53 | 0.18 |
| 4557743 | pyrin isoform 1 | 0.014 | 1.61 | 0.21 |
| 4758756 | nucleosome assembly protein 1-like 1 | 0.014 | 0.39 | -0.41 |
| 8923532 | ceroid-lipofuscinosis neuronal protein 6 | 0.014 | 0.55 | -0.26 |
| 78000183 | 60S ribosomal protein L14 | 0.014 | 0.46 | -0.34 |
| 4505893 | proteolipid protein 2 | 0.014 | 1.30 | 0.11 |
| 112789550 | poly [ADP-ribose] polymerase 4 | 0.015 | 1.65 | 0.22 |
| 74136552 | LDLR chaperone MESD precursor | 0.015 | 0.59 | -0.23 |
| 38348436 | carbonic anhydrase 13 | 0.015 | 0.43 | -0.37 |
| 40317626 | thrombospondin-1 precursor | 0.015 | 0.71 | -0.15 |
| 66346698 | alpha-N-acetylglucosaminidase precursor | 0.015 | 0.49 | -0.31 |
| 4506411 | ran GTPase-activating protein 1 | 0.015 | 1.55 | 0.19 |
| 4506013 | protein phosphatase 1 regulatory subunit 7 | 0.015 | 0.64 | -0.19 |
| 4758256 | eukaryotic translation initiation factor 2 subunit 1 | 0.015 | 1.31 | 0.12 |
| 4758772 | NADH dehydrogenase [ubiquinone] 1 alpha subcomplex subunit 3 | 0.015 | 1.58 | 0.20 |
| 16306548 | serine--tRNA ligase, cytoplasmic | 0.015 | 0.68 | -0.17 |
| 207452735 | epiplakin | 0.016 | 1.56 | 0.19 |
| 11055992 | retinoid-inducible serine carboxypeptidase precursor | 0.016 | 1.52 | 0.18 |
| 209862760 | NADH dehydrogenase [ubiquinone] 1 beta subcomplex subunit 11, mitochondrial isoform 2 | 0.016 | 1.64 | 0.21 |
| 23397666 | paired amphipathic helix protein Sin3a | 0.016 | 1.82 | 0.26 |
| 7019569 | vacuolar protein sorting-associated protein 4A | 0.016 | 0.57 | -0.25 |
| 7706481 | calcium-binding protein 39 | 0.016 | 0.65 | -0.19 |
| 14702171 | DNA-directed RNA polymerase II subunit RPB3 | 0.016 | 2.11 | 0.32 |
| 83376130 | elongation factor 1-beta | 0.016 | 0.53 | -0.28 |
| 4758476 | GRB2-related adapter protein 2 | 0.016 | 0.73 | -0.14 |
| 4826972 | RNA-binding protein 8A | 0.016 | 2.44 | 0.39 |
| 13904866 | 60S ribosomal protein L28 isoform 2 | 0.016 | 1.85 | 0.27 |
| 171460997 | CD226 antigen precursor | 0.017 | 0.45 | -0.35 |
| 4758460 | platelet glycoprotein V precursor | 0.017 | 0.49 | -0.31 |
| 71979937 | leukosialin precursor | 0.017 | 1.56 | 0.19 |
| 53759148 | retinoblastoma-binding protein 5 isoform 1 | 0.017 | 0.62 | -0.21 |
| 4757766 | rho GTPase-activating protein 1 | 0.017 | 1.31 | 0.12 |
| 371874557 | sorting nexin-12 isoform 1 | 0.017 | 2.17 | 0.34 |
| 17865802 | vacuolar protein sorting-associated protein 4B | 0.017 | 0.69 | -0.16 |
| 13775602 | NAD-dependent protein deacetylase sirtuin-2 isoform 2 | 0.017 | 0.43 | -0.37 |
| 50345991 | ATP synthase subunit delta, mitochondrial precursor | 0.017 | 1.78 | 0.25 |
| 147904340 | WD repeat-containing protein 82 | 0.017 | 2.50 | 0.40 |
| 151101191 | interferon-induced transmembrane protein 2 | 0.017 | 2.43 | 0.38 |
| 5031887 | lipoma-preferred partner isoform a | 0.017 | 1.54 | 0.19 |
| 8923427 | OCIA domain-containing protein 1 isoform 1 | 0.017 | 2.00 | 0.30 |
| 14591909 | 60S ribosomal protein L5 | 0.017 | 1.46 | 0.16 |
| 194239729 | elongation factor 1-delta isoform 4 | 0.018 | 0.63 | -0.20 |
| 23510389 | myotubularin-related protein 3 isoform b | 0.018 | 0.68 | -0.17 |
| 154813199 | poly [ADP-ribose] polymerase 14 | 0.018 | 1.78 | 0.25 |
| 4758874 | transmembrane 9 superfamily member 2 precursor | 0.018 | 0.65 | -0.19 |
| 42490749 | ATP-binding cassette sub-family B member 7, mitochondrial isoform 1 | 0.018 | 0.55 | -0.26 |
| 171460914 | dynamin-1-like protein isoform 1 | 0.019 | 0.68 | -0.17 |
| 4758762 | asparagine--tRNA ligase, cytoplasmic | 0.019 | 0.58 | -0.24 |
| 11386157 | cytidine deaminase | 0.019 | 0.49 | -0.31 |
| 4507231 | single-stranded DNA-binding protein, mitochondrial precursor | 0.019 | 1.51 | 0.18 |
| 19923592 | oxysterol-binding protein-related protein 11 | 0.019 | 0.53 | -0.28 |
| 7705431 | translation machinery-associated protein 7 | 0.019 | 1.69 | 0.23 |
| 385298707 | hippocalcin-like protein 1 | 0.020 | 0.39 | -0.41 |
| 4507129 | small nuclear ribonucleoprotein E | 0.020 | 0.49 | -0.31 |
| 40789233 | ubiquinone biosynthesis protein COQ9, mitochondrial precursor | 0.020 | 1.77 | 0.25 |
| 10092657 | NADH dehydrogenase [ubiquinone] 1 alpha subcomplex subunit 12 isoform a | 0.020 | 1.76 | 0.24 |
| 4758440 | glia maturation factor gamma | 0.020 | 1.72 | 0.24 |
| 168229174 | RNA-binding protein 27 | 0.020 | 2.30 | 0.36 |
| 4557233 | short-chain specific acyl-CoA dehydrogenase, mitochondrial precursor | 0.020 | 1.49 | 0.17 |
| 4506029 | serine/threonine-protein phosphatase 6 catalytic subunit isoform b | 0.020 | 0.53 | -0.27 |
| 29826294 | Golgi reassembly-stacking protein 2 isoform 1 | 0.021 | 1.66 | 0.22 |
| 47132620 | keratin, type II cytoskeletal 2 epidermal | 0.021 | 1.49 | 0.17 |
| 4506707 | 40S ribosomal protein S25 | 0.021 | 1.51 | 0.18 |
| 304555583 | elongation factor 1-delta isoform 1 | 0.021 | 0.64 | -0.19 |
| 5729980 | phosphomevalonate kinase | 0.021 | 1.40 | 0.15 |
| 24430132 | WW domain-binding protein 2 | 0.021 | 1.44 | 0.16 |
| 4502049 | aldose reductase | 0.021 | 1.39 | 0.14 |
| 285002233 | glycerol-3-phosphate dehydrogenase, mitochondrial precursor | 0.021 | 1.40 | 0.15 |
| 83320070 | gasdermin-D | 0.021 | 0.56 | -0.25 |
| 21396480 | RNA-binding protein Raly isoform 2 | 0.022 | 1.50 | 0.18 |
| 5901908 | butyrophilin subfamily 3 member A3 isoform a precursor | 0.022 | 0.63 | -0.20 |
| 124494254 | proliferation-associated protein 2G4 | 0.022 | 1.30 | 0.11 |
| 94721347 | heparanase isoform 1 preproprotein | 0.022 | 0.63 | -0.20 |
| 32189392 | peroxiredoxin-2 | 0.022 | 1.36 | 0.13 |
| 4503015 | copine-3 | 0.022 | 0.61 | -0.21 |
| 238776833 | THO complex subunit 4 | 0.022 | 0.71 | -0.15 |
| 4758442 | glia maturation factor beta | 0.022 | 1.82 | 0.26 |
| 33946297 | DBIRD complex subunit ZNF326 isoform 1 | 0.022 | 0.45 | -0.34 |
| 388454220 | CCR4-NOT transcription complex subunit 1 isoform c | 0.023 | 1.51 | 0.18 |
| 67514036 | SH3 domain-containing kinase-binding protein 1 isoform b | 0.023 | 1.49 | 0.17 |
| 4505939 | DNA-directed RNA polymerase II subunit RPB1 | 0.023 | 1.68 | 0.22 |
| 110815813 | ankyrin repeat and FYVE domain-containing protein 1 isoform 1 | 0.023 | 0.63 | -0.20 |
| 45827771 | enhancer of mRNA-decapping protein 4 | 0.023 | 0.69 | -0.16 |
| 21361619 | toll-interacting protein | 0.023 | 1.95 | 0.29 |
| 55956899 | keratin, type I cytoskeletal 9 | 0.023 | 1.58 | 0.20 |
| 150378549 | EH domain-binding protein 1-like protein 1 | 0.024 | 0.22 | -0.66 |
| 4758384 | peptidyl-prolyl cis-trans isomerase FKBP5 isoform 1 | 0.024 | 1.58 | 0.20 |
| 114842389 | myosin-7B | 0.024 | 5.63 | 0.75 |
| 199559805 | phostensin | 0.024 | 1.71 | 0.23 |
| 116812620 | protein FAM98C | 0.024 | 0.41 | -0.39 |
| 4505317 | protein phosphatase 1 regulatory subunit 12A isoform a | 0.024 | 1.32 | 0.12 |
| 288557345 | MOB kinase activator 2 isoform 1 | 0.024 | 1.67 | 0.22 |
| 4502517 | carbonic anhydrase 1 | 0.025 | 2.14 | 0.33 |
| 13027378 | glucosamine-6-phosphate isomerase 1 | 0.025 | 1.54 | 0.19 |
| 56699465 | protein-tyrosine sulfotransferase 2 precursor | 0.025 | 0.44 | -0.35 |
| 256017247 | phospholipase DDHD2 isoform 1 | 0.025 | 0.47 | -0.33 |
| 259013553 | 2-oxoglutarate dehydrogenase, mitochondrial isoform 3 precursor | 0.025 | 1.35 | 0.13 |
| 148225659 | endonuclease domain-containing 1 protein precursor | 0.025 | 0.73 | -0.14 |
| 27262630 | nuclear autoantigenic sperm protein isoform 3 | 0.025 | 1.71 | 0.23 |
| 304434690 | serine/threonine-protein phosphatase 6 regulatory ankyrin repeat subunit B isoform A | 0.025 | 1.32 | 0.12 |
| 6005757 | FACT complex subunit SPT16 | 0.025 | 1.48 | 0.17 |
| 83641876 | probable serine carboxypeptidase CPVL precursor | 0.025 | 1.73 | 0.24 |
| 4501867 | aconitate hydratase, mitochondrial precursor | 0.025 | 1.36 | 0.13 |
| 4506191 | proteasome subunit beta type-10 precursor | 0.025 | 1.38 | 0.14 |
| 13562114 | tubulin beta-1 chain | 0.026 | 0.68 | -0.17 |
| 410170253 | PREDICTED: HLA class II histocompatibility antigen, DQ beta 1 chain-like | 0.026 | 1.93 | 0.29 |
| 4504035 | GMP synthase [glutamine-hydrolyzing] | 0.026 | 0.37 | -0.44 |
| 11321585 | guanine nucleotide-binding protein G(I)/G(S)/G(T) subunit beta-1 | 0.026 | 1.35 | 0.13 |
| 164519136 | endothelin-converting enzyme 1 isoform 3 | 0.026 | 0.60 | -0.22 |
| 187608516 | arf-GAP with coiled-coil, ANK repeat and PH domain-containing protein 2 | 0.026 | 1.51 | 0.18 |
| 449083351 | protein transport protein Sec16A isoform 2 | 0.027 | 1.41 | 0.15 |
| 28872738 | 39S ribosomal protein L43, mitochondrial isoform d | 0.027 | 1.55 | 0.19 |
| 5031877 | lamin-B1 isoform 1 | 0.027 | 1.49 | 0.17 |
| 212286179 | gamma-parvin | 0.027 | 1.61 | 0.21 |
| 6466466 | ran-binding protein 3 isoform RANBP3-d | 0.027 | 1.94 | 0.29 |
| 324711029 | arachidonate 5-lipoxygenase-activating protein isoform 2 | 0.027 | 1.64 | 0.21 |
| 5453906 | CDP-diacylglycerol--inositol 3-phosphatidyltransferase | 0.027 | 0.29 | -0.53 |
| 51242941 | lymphocyte antigen 6 complex locus protein G6f precursor | 0.027 | 0.51 | -0.29 |
| 4506605 | 60S ribosomal protein L23 | 0.027 | 2.19 | 0.34 |
| 4505061 | cation-dependent mannose-6-phosphate receptor isoform 1 precursor | 0.028 | 0.48 | -0.32 |
| 32699045 | Parkinson disease 7 domain-containing protein 1 precursor | 0.028 | 0.54 | -0.27 |
| 116875844 | phosphopantothenate--cysteine ligase isoform a | 0.028 | 1.44 | 0.16 |
| 157412280 | set1/Ash2 histone methyltransferase complex subunit ASH2 isoform a | 0.028 | 0.60 | -0.22 |
| 94536842 | ribose-5-phosphate isomerase | 0.028 | 1.61 | 0.21 |
| 4506613 | 60S ribosomal protein L22 proprotein | 0.028 | 1.62 | 0.21 |
| 42734503 | caprin-1 isoform 2 | 0.028 | 1.62 | 0.21 |
| 96975097 | ras-related protein Rab-6B | 0.028 | 0.69 | -0.16 |
| 61175258 | cytochrome c oxidase assembly factor 6 homolog isoform 1 | 0.028 | 1.47 | 0.17 |
| 9845516 | protein S100-A4 | 0.029 | 0.67 | -0.18 |
| 56682964 | legumain preproprotein | 0.029 | 1.72 | 0.24 |
| 10835063 | nucleophosmin isoform 1 | 0.029 | 1.59 | 0.20 |
| 262118328 | leukocyte-specific transcript 1 protein isoform 4 | 0.029 | 1.88 | 0.27 |
| 12408677 | prefoldin subunit 4 | 0.029 | 0.57 | -0.25 |
| 4504193 | transcription initiation factor IIB | 0.029 | 0.66 | -0.18 |
| 6678271 | TAR DNA-binding protein 43 | 0.030 | 1.46 | 0.16 |
| 116875826 | HD domain-containing protein 2 | 0.030 | 1.61 | 0.21 |
| 315113895 | biogenesis of lysosome-related organelles complex 1 subunit 1 | 0.030 | 2.04 | 0.31 |
| 11641247 | Golgi-associated plant pathogenesis-related protein 1 | 0.030 | 1.52 | 0.18 |
| 8923040 | CDKN2A-interacting protein | 0.030 | 2.01 | 0.30 |
| 154800489 | erlin-1 | 0.030 | 1.60 | 0.20 |
| 296531406 | ES1 protein homolog, mitochondrial isoform Ia precursor | 0.030 | 0.61 | -0.22 |
| 31982936 | sphingosine-1-phosphate lyase 1 | 0.030 | 1.80 | 0.26 |
| 68800430 | HCLS1-binding protein 3 | 0.031 | 1.66 | 0.22 |
| 115527097 | serine/threonine-protein kinase MRCK beta | 0.031 | 0.50 | -0.30 |
| 47519616 | tropomyosin beta chain isoform 2 | 0.031 | 0.48 | -0.32 |
| 289577109 | ADP-ribosylation factor-binding protein GGA3 isoform 1 | 0.031 | 1.83 | 0.26 |
| 155030185 | protein asunder homolog | 0.031 | 0.40 | -0.40 |
| 124248539 | axin interactor, dorsalization-associated protein | 0.031 | 0.65 | -0.18 |
| 194018550 | C-Maf-inducing protein isoform C-Mip | 0.031 | 0.73 | -0.14 |
| 4504445 | heterogeneous nuclear ribonucleoprotein A1 isoform a | 0.031 | 1.70 | 0.23 |
| 156564403 | pyruvate dehydrogenase E1 component subunit beta, mitochondrial isoform 1 precursor | 0.032 | 1.31 | 0.12 |
| 5803187 | transaldolase | 0.032 | 1.30 | 0.12 |
| 11342670 | azurocidin preproprotein | 0.032 | 1.52 | 0.18 |
| 38788353 | ragulator complex protein LAMTOR5 | 0.032 | 0.60 | -0.22 |
| 13904870 | 40S ribosomal protein S5 | 0.032 | 0.58 | -0.24 |
| 193083197 | ubiquitin-like domain-containing CTD phosphatase 1 | 0.032 | 2.10 | 0.32 |
| 4506773 | protein S100-A9 | 0.032 | 0.63 | -0.20 |
| 11342676 | cdc42-interacting protein 4 | 0.033 | 0.28 | -0.56 |
| 4885287 | guanine nucleotide-binding protein G(I)/G(S)/G(O) subunit gamma-5 precursor | 0.033 | 1.46 | 0.17 |
| 19923665 | BRI3-binding protein precursor | 0.033 | 2.27 | 0.36 |
| 67763816 | guanylate cyclase soluble subunit alpha-3 isoform A | 0.033 | 0.50 | -0.30 |
| 315139026 | serine/threonine-protein kinase TAO1 isoform 2 | 0.033 | 1.56 | 0.19 |
| 70906439 | fibrinogen gamma chain isoform gamma-B precursor | 0.033 | 0.62 | -0.21 |
| 7706501 | WW domain-binding protein 11 | 0.033 | 1.49 | 0.17 |
| 38202214 | protein transport protein Sec23A | 0.034 | 1.34 | 0.13 |
| 7305303 | nck-associated protein 1 isoform 1 | 0.034 | 1.87 | 0.27 |
| 47271443 | serine/arginine-rich splicing factor 2 | 0.034 | 1.33 | 0.12 |
| 23943920 | cytosolic phospholipase A2 | 0.034 | 0.68 | -0.17 |
| 4504327 | trifunctional enzyme subunit beta, mitochondrial precursor | 0.034 | 1.40 | 0.15 |
| 21361565 | ATP synthase subunit b, mitochondrial precursor | 0.035 | 1.49 | 0.17 |
| 4557581 | fatty acid-binding protein, epidermal | 0.035 | 1.80 | 0.26 |
| 62420875 | integrin-linked protein kinase isoform 1 | 0.035 | 0.73 | -0.14 |
| 17402900 | far upstream element-binding protein 1 | 0.035 | 1.63 | 0.21 |
| 150378533 | ubiquitin carboxyl-terminal hydrolase 7 | 0.035 | 1.71 | 0.23 |
| 32129199 | SAP domain-containing ribonucleoprotein | 0.036 | 1.40 | 0.15 |
| 58197556 | putative transferase CAF17, mitochondrial precursor | 0.036 | 1.30 | 0.12 |
| 29826321 | alpha-adducin isoform b | 0.036 | 1.45 | 0.16 |
| 38093659 | stimulator of interferon genes protein | 0.036 | 0.61 | -0.22 |
| 269847422 | major facilitator superfamily domain-containing protein 1 isoform 2 | 0.036 | 1.52 | 0.18 |
| 309384267 | oxidation resistance protein 1 isoform 4 | 0.037 | 0.69 | -0.16 |
| 124244088 | BRO1 domain-containing protein BROX | 0.037 | 1.36 | 0.13 |
| 116805340 | glycine--tRNA ligase precursor | 0.037 | 1.47 | 0.17 |
| 91718899 | mitogen-activated protein kinase 3 isoform 1 | 0.037 | 1.61 | 0.21 |
| 8393009 | vacuolar protein sorting-associated protein 51 homolog | 0.037 | 1.51 | 0.18 |
| 194097354 | eukaryotic peptide chain release factor GTP-binding subunit ERF3A isoform 2 | 0.038 | 1.42 | 0.15 |
| 27436901 | 39S ribosomal protein L12, mitochondrial | 0.038 | 1.63 | 0.21 |
| 4507555 | thymopoietin isoform alpha | 0.038 | 1.34 | 0.13 |
| 47933341 | phosphotriesterase-related protein isoform 1 | 0.038 | 1.35 | 0.13 |
| 14702162 | CAP-Gly domain-containing linker protein 2 isoform 2 | 0.039 | 1.35 | 0.13 |
| 115334675 | protein FAM105B | 0.039 | 1.99 | 0.30 |
| 56181387 | E3 ubiquitin-protein ligase CHIP | 0.039 | 0.61 | -0.21 |
| 40548322 | rho GTPase-activating protein 27 isoform a | 0.039 | 1.47 | 0.17 |
| 132626790 | low density lipoprotein receptor adapter protein 1 | 0.039 | 0.74 | -0.13 |
| 4505733 | platelet factor 4 precursor | 0.039 | 0.45 | -0.34 |
| 5174409 | CD2 antigen cytoplasmic tail-binding protein 2 | 0.039 | 2.16 | 0.33 |
| 4826848 | NADH dehydrogenase [ubiquinone] 1 alpha subcomplex subunit 5 | 0.040 | 0.60 | -0.22 |
| 98986457 | host cell factor 1 | 0.040 | 2.08 | 0.32 |
| 14150147 | protein syndesmos isoform 1 | 0.040 | 1.64 | 0.21 |
| 5031703 | ras GTPase-activating protein-binding protein 1 | 0.040 | 0.67 | -0.17 |
| 5902090 | solute carrier family 2, facilitated glucose transporter member 3 | 0.040 | 0.66 | -0.18 |
| 38679892 | peptidyl-prolyl cis-trans isomerase NIMA-interacting 4 isoform 1 | 0.040 | 1.46 | 0.17 |
| 27597059 | dnaJ homolog subfamily C member 9 | 0.040 | 0.59 | -0.23 |
| 95147555 | microtubule-associated protein 1A | 0.040 | 1.62 | 0.21 |
| 41393614 | ras-related protein Rab-5C isoform a | 0.041 | 1.39 | 0.14 |
| 54114974 | guanine nucleotide-binding protein G(I)/G(S)/G(O) subunit gamma-2 precursor | 0.041 | 1.48 | 0.17 |
| 25777615 | 26S proteasome non-ATPase regulatory subunit 7 | 0.041 | 1.57 | 0.19 |
| 154146191 | heat shock protein HSP 90-alpha isoform 2 | 0.041 | 1.36 | 0.13 |
| 17978477 | vacuolar protein sorting-associated protein 11 homolog | 0.041 | 1.30 | 0.11 |
| 7657381 | pre-mRNA-processing factor 19 | 0.041 | 1.62 | 0.21 |
| 148233642 | transmembrane protein C16orf54 | 0.042 | 2.34 | 0.37 |
| 24497451 | nuclear pore complex protein Nup50 isoform b | 0.042 | 1.47 | 0.17 |
| 5031707 | leucine-rich repeat-containing protein 32 precursor | 0.042 | 1.42 | 0.15 |
| 7382480 | rho GTPase-activating protein 6 isoform 4 | 0.042 | 0.73 | -0.14 |
| 28178821 | isocitrate dehydrogenase [NAD] subunit beta, mitochondrial isoform a precursor | 0.042 | 2.17 | 0.34 |
| 4826870 | nucleobindin-2 precursor | 0.043 | 0.61 | -0.21 |
| 24308201 | adipocyte plasma membrane-associated protein | 0.043 | 1.31 | 0.12 |
| 4506695 | 40S ribosomal protein S19 | 0.043 | 1.42 | 0.15 |
| 224591399 | UPF0688 protein C1orf174 | 0.043 | 1.47 | 0.17 |
| 38176151 | protein lunapark | 0.043 | 1.31 | 0.12 |
| 217272894 | 116 kDa U5 small nuclear ribonucleoprotein component isoform b | 0.043 | 1.47 | 0.17 |
| 345842414 | serine/threonine-protein phosphatase 2B catalytic subunit gamma isoform isoform 3 | 0.043 | 1.32 | 0.12 |
| 6005721 | erlin-2 isoform 1 | 0.043 | 1.81 | 0.26 |
| 347659028 | glutamine--fructose-6-phosphate aminotransferase [isomerizing] 1 isoform 1 | 0.044 | 1.33 | 0.12 |
| 145580600 | schlafen family member 5 | 0.044 | 1.59 | 0.20 |
| 13027602 | DDRGK domain-containing protein 1 precursor | 0.044 | 1.77 | 0.25 |
| 4502753 | cyclin-dependent kinase 4 inhibitor D | 0.044 | 0.59 | -0.23 |
| 27597085 | tropomyosin alpha-1 chain isoform 5 | 0.044 | 0.53 | -0.28 |
| 30795227 | D-tyrosyl-tRNA(Tyr) deacylase 1 | 0.044 | 1.56 | 0.19 |
| 24797076 | HLA class II histocompatibility antigen, DP beta 1 chain precursor | 0.044 | 1.50 | 0.18 |
| 5453543 | aldo-keto reductase family 1 member C1 | 0.044 | 0.61 | -0.22 |
| 4503499 | eukaryotic translation initiation factor 1A, X-chromosomal | 0.044 | 1.31 | 0.12 |
| 372266173 | ras-related protein Rab-34 isoform 7 | 0.045 | 0.63 | -0.20 |
| 21361418 | ras-related protein Rab-30 | 0.045 | 0.63 | -0.20 |
| 5453539 | multifunctional protein ADE2 isoform 2 | 0.045 | 1.58 | 0.20 |
| 5453676 | granzyme A precursor | 0.045 | 1.63 | 0.21 |
| 51599156 | chromodomain-helicase-DNA-binding protein 4 | 0.045 | 1.73 | 0.24 |
| 13994151 | PDZ and LIM domain protein 1 | 0.045 | 0.66 | -0.18 |
| 4506205 | proteasome subunit beta type-9 proprotein | 0.045 | 1.68 | 0.23 |
| 50428938 | ATPase ASNA1 | 0.045 | 0.63 | -0.20 |
| 19743823 | integrin beta-1 isoform 1A precursor | 0.046 | 0.57 | -0.24 |
| 112293277 | dnaJ homolog subfamily C member 8 | 0.046 | 1.50 | 0.17 |
| 222352151 | poly(rC)-binding protein 1 | 0.046 | 1.39 | 0.14 |
| 73611906 | phosphorylase b kinase regulatory subunit beta isoform b | 0.046 | 0.64 | -0.19 |
| 507834126 | ADP-ribosylation factor-like protein 6-interacting protein 4 isoform 3 | 0.046 | 2.20 | 0.34 |
| 21704263 | protein max isoform b | 0.046 | 1.48 | 0.17 |
| 5031873 | protein ERGIC-53 precursor | 0.046 | 1.36 | 0.13 |
| 78000213 | DNA methyltransferase 1-associated protein 1 | 0.046 | 2.05 | 0.31 |
| 4504007 | glycerol kinase isoform b | 0.047 | 0.70 | -0.16 |
| 94681038 | phospholipase D4 | 0.047 | 2.22 | 0.35 |
| 4507909 | wiskott-Aldrich syndrome protein | 0.047 | 1.36 | 0.13 |
| 7706322 | UPF0568 protein C14orf166 | 0.047 | 1.75 | 0.24 |
| 151108473 | mitochondrial fission 1 protein | 0.047 | 1.48 | 0.17 |
| 7661920 | eukaryotic initiation factor 4A-III | 0.047 | 1.39 | 0.14 |
| 296939604 | serine/threonine-protein kinase WNK1 isoform 2 | 0.047 | 1.46 | 0.16 |
| 7661744 | basic leucine zipper and W2 domain-containing protein 2 | 0.047 | 0.55 | -0.26 |
| 49574532 | glycogen synthase kinase-3 alpha | 0.048 | 0.63 | -0.20 |
| 15431290 | 60S ribosomal protein L11 isoform 1 | 0.048 | 1.70 | 0.23 |
| 28173554 | histone H2B type 3-B | 0.048 | 1.35 | 0.13 |
| 7706339 | peptidyl-prolyl cis-trans isomerase-like 1 | 0.048 | 1.44 | 0.16 |
| 155030240 | CCA tRNA nucleotidyltransferase 1, mitochondrial | 0.048 | 0.57 | -0.24 |
| 4503475 | elongation factor 1-alpha 2 | 0.048 | 1.52 | 0.18 |
| 313661428 | serine/threonine-protein kinase Nek1 isoform 3 | 0.048 | 0.69 | -0.16 |
| 89903012 | cell division control protein 42 homolog isoform 1 precursor | 0.048 | 1.59 | 0.20 |
| 89191868 | von Willebrand factor preproprotein | 0.048 | 0.71 | -0.15 |
| 7706667 | trafficking protein particle complex subunit 4 | 0.048 | 1.91 | 0.28 |
| 21614544 | protein S100-A8 | 0.049 | 0.59 | -0.23 |
| 13124873 | core-binding factor subunit beta isoform 2 | 0.049 | 1.32 | 0.12 |
| 217035154 | capZ-interacting protein | 0.049 | 1.42 | 0.15 |
| 13489085 | ubiquitin-conjugating enzyme E2 G1 | 0.049 | 1.47 | 0.17 |
| 28195394 | histone H2A type 2-B | 0.049 | 1.45 | 0.16 |
| 4506027 | serine/threonine-protein phosphatase 4 catalytic subunit | 0.049 | 1.37 | 0.14 |
| 5031741 | dnaJ homolog subfamily A member 2 | 0.049 | 1.76 | 0.24 |
| 70780353 | ankyrin-1 isoform 4 | 0.049 | 1.48 | 0.17 |
| 4505181 | microsomal glutathione S-transferase 2 isoform 1 precursor | 0.049 | 1.62 | 0.21 |
| 313569822 | RPL17-C18orf32 protein isoform 1 | 0.049 | 1.93 | 0.29 |
| 4503843 | AP-1 complex subunit gamma-like 2 | 0.049 | 0.71 | -0.15 |
| 19923260 | ras-related protein Rab-4A isoform 1 | 0.050 | 0.65 | -0.18 |
| 117938251 | bcl-2-associated transcription factor 1 isoform 2 | 0.050 | 2.27 | 0.36 |

**Figure S1**: Functional annotation clusters generated from proteins with relatively higher abundance in all ME/CFS versus all controls (n = 38, P < 0.01, Log_10_(Fold-Change) > 0.2). Cluster **A** has an enrichment score = 4.01, cluster **B** enrichment score = 2.54, and cluster **C** enrichment score = 1.32. The enrichment score is the geometric mean (in -log scale) of member's P values in a corresponding annotation cluster and is used to rank their biological significance, with the top ranked annotation groups having consistent lower P-values for their annotation members. Cluster **A** involves histone methylation, cluster **B** WD repeat domains, and cluster **C** protein catabolism and ubiquination.

**A**

**B**

**C**


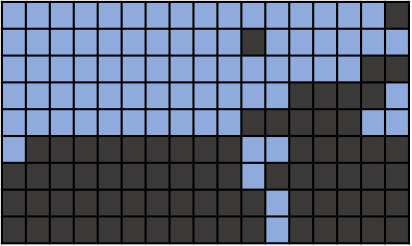


Histone cluster 1 H1 family member c (HIST1H1C)

Histone cluster 1 H1 family member d (HIST1H1D)

Histone cluster 1 H1 family member e (HIST1H1E)

Histone cluster 1 H1 family member b (HIST1H1B)

Histone cluster 1 H1 family member a (HIST1H1A)

Cullin 3 (CUL3)

Serine’/threonine kinase receptor associated protein (STRAP)

Adhesion regulating molecule 1 (ADRH1)

Splicing factor 3a subunit 1 (SF3A1)

IPR011991:Winged helix-turn-helix DNA-binding domain

IPR005818: Histone H1/H5

GO:0031490~chromatin DNA binding

Citrullination

IPR005819: Histone H5

GO:0000786~nucleosome

GO:0006334~nucleosome assembly

Chromosome

DNA-binding

GO:0000122~negative regulation of transcription from RNA polymerase II promoter

Isopeptide bond

GO:0098532~histone H3-K27 trimethylation

GO:0016584~nucleosome positioning

GO:0080182~histone H3-K4 trimethylation

GO:0005719~nuclear euchromatin

GO:0000798~nuclear chromatin


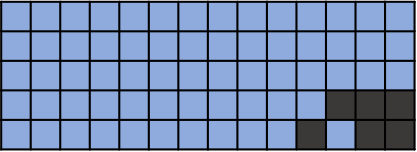


Repeat: WD 4

Repeat: WD 3

SM00320: WD40

WD repeat

Repeat: WD 1

Repeat: WD 2

IPR001680: WD40 repeat

IPR017986: WD40-repeat-containing domain

IPR015943: WD40/YVTN repeat-like-containing domain

Repeat: WD 5

IPR019775: WD40 repeat, conserved site

Repeat: WD 6

IPR020472: G-protein beta WD-40 repeat

Repeat: WD 7

WD repeat domain 37 (WDR37)

PWP2 periodic tryptophan protein homolog (PWP2)

Serine/threonine kinase receptor associated protein (STRAP)

WD repeat domain 77 (WDR77)

Actin related protein 2/3 complex subunit 1A (ARPC1A)


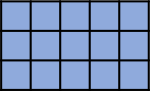


Cullin 3 (CUL3)

Proteasome 26S subunit, non-ATPase 14 (PSMD14)

Proteasome subunit beta 4 (PSMB4)

GO:0031145~anaphase-promoting complex-dependent catabolic process

GO:0090090~negative regulation of canonical Wnt signalling pathway

GO:0000289~protein polyubiquination

GO:0043161~proteasome-mediated ubiquitin-dependent protein catabolic process

GO:0000165~MAPK cascade


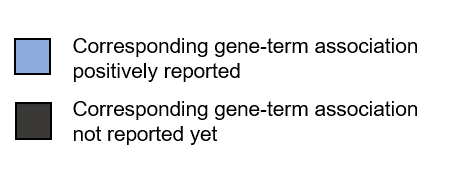


**Figure S2:** Functional annotation clusters generated from proteins with relatively higher abundance in all ME/CFS thversus all controls (n = 38, P< 0.01, Log_10_(Fold-Change) > 0.2). Cluster **D** has an enrichment score = 1.3, cluster **E** enrichment score = 1.02, and cluster **F** enrichment score = 0.9. The enrichment score is the geometric mean (in -log scale) of member's P-values in a corresponding annotation cluster and is used to rank their biological significance, with the top ranked annotation groups having consistent lower P-values for their annotation members. Cluster **D** involves NAD and NAD(P) binding, cluster **E** the Golgi apparatus and cluster **F** the Mitochondrion.

**D**

**E**

**F**


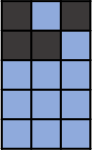

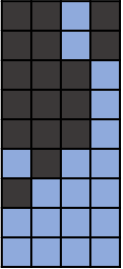

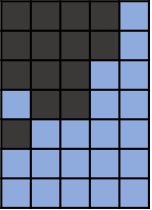


SUMO1 activating enzyme subunit 1 (SAE1)

Acetyl-CoA acetyltransferase 2 (ACAT2)

Inositol-3-phosphate synthase 1 (ISYNA1)

3-hydroxybutyrate dehydrogenase, type 1 (BDH1)

Glyceraldehyde-3-phosphate dehydrogenase, spermatogenic (GAPDHS)

NAD

IPR016040: NAD(P)-binding domain

hsa01100:Metabolic pathways

WD repeat domain 77 (WDR77)

Proteasome assembly chaperone 1 (PSMG1)

Solute carrier family 25 member 3 (SLC25A3)

Solute carrier family 25 member 11 (SLC25A11)

Nuclear RNA export factor 1 (NXF1)

Vesicle transport through interaction with t-SNAREs 1B (VTI1B)

Chloride channel CLIC like 1 (CLCC1)

ADP ribosylation factor 4 (ARF4)

Cullin 3 (CUL3)

GO:0006888~ER to Golgi vesicle-mediated transport

Golgi apparatus

GO:0005794~Golgi apparatus

Transport

Acetyl-CoA acetyltransferase 2 (ACAT2)

FUN14 domain containing 2 (FUNDC2)

Up-regulated during skeletal muscle growth 5 homolog (USMG5)

Solute carrier family 25 member 11 (SLC25A11)

Thioredoxin reductase 2 (TXNRD2)

3-hyroxybutyrate dehydrogenase, type 1 (BDH1)

Solute carrier family 25 member 2 (SLC25A3)

Mitochondrion inner membrane

Transit peptide:Mitochondrion

Transit peptide

Mitochondrion

GO:0005739~mitochondrion


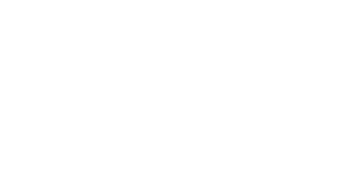


**A**

**B**

**D**


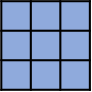


Calnexin (CANX)

Peptidylprolyl isomerase A (PPIA)

Dna J heat shock protein family (Hsp40) member B11 (DNAJB11)

GO:0006457~protein folding

GO:0016020~membrane

GO:0051082~unfolded protein binding


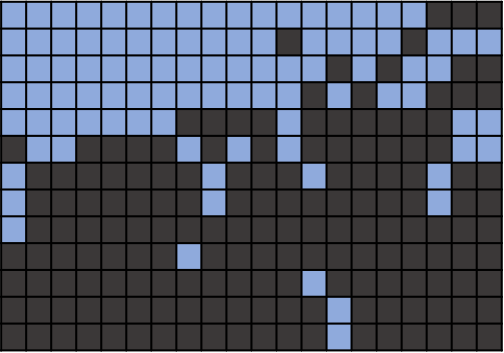


Glycoprotein Ib platelet beta subunit (GP1BB)

Chromosome 6 open reading frame 25 (C6orf25)

Plexin A4 (PLXN4)

Glycoprotein IX platelet (GP9)

Calnexin (CANX)

DnaJ heat shock protein family (Hsp40) member B11 (DNAJB11)

Src like adaptor 2 (SLA2)

Copine 2 (CPNE2)

ADP ribosylation factor like GTPase 3 (ARL3)

Peptidylprolyl isomerase A (PPIA)

RAS p21 protein activator 1 (RASA1)

Hemoglobin subunit gamma 2 (HBG2)

Hemoglobin subunit delta (HBD)

Membrane

Signal

Signal peptide

Topological domain:Cytoplasmic

Transmembrane region

Transmembrane helix

Transmembrane

Glycoprotein

GO:0005886~plasma membrane

Glycosylation site:N-linked (GlcNAc…)

Topologial domain:Extracellular

Disulfide bond

Splice variant

GO;0007596~blood coagulation

GO:0016021~integral component of membrane

GO:0030168~platelet activation

GO:0005887~integral component of plasma membrane

Cell membrane

GO:0005783~endoplasmic reticulum

Endoplasmic reticulum


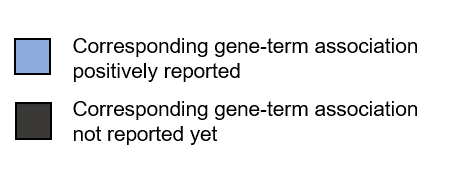


**Figure S3:** Functional annotation clusters generated from proteins with relatively lower abundance in all ME/CFS versus all controls (n = 22, *P* < 0.01, Log_10_(Fold-Change) < -0.2). Cluster A has an enrichment score = 1.4, cluster B enrichment score = 0.61. The enrichment score is the geometric mean (in -log scale) of member's p-values in a corresponding annotation cluster and is used to rank their biological significance, with the top ranked annotation groups having consistent lower p-values for their annotation members. Cluster **A** involves protein folding and cluster **B** blood coagulation and platelet activation.
